# Highly Efficient Van Der Waals Heterojunction on Graphdiyne toward the High‐Performance Photodetector

**DOI:** 10.1002/advs.202300925

**Published:** 2023-07-09

**Authors:** Dinh Phuc Do, Chengyun Hong, Viet Q Bui, Thi Hue Pham, Sohyeon Seo, Van Dam Do, Thanh Luan Phan, Kim My Tran, Surajit Haldar, Byung‐wook Ahn, Seong Chu Lim, Woo Jong Yu, Seong‐Gon Kim, Ji‐Hee Kim, Hyoyoung Lee

**Affiliations:** ^1^ Department of Chemistry Sungkyunkwan University Suwon 16419 Republic of Korea; ^2^ Department of Energy Science Sungkyunkwan University Suwon 16419 Republic of Korea; ^3^ Advanced Institute of Science and Technology The University of Danang 41 Le Duan Danang 92026 Vietnam; ^4^ Creative Research Institute Sungkyunkwan University Suwon 16419 Republic of Korea; ^5^ Department of Electrical and Computer Engineering Sungkyunkwan University Suwon 16419 Republic of Korea; ^6^ Department of Physics and Astronomy and Center for Computational Sciences Mississippi State University Mississippi State MS 39762 USA; ^7^ Department of Biophysics Sungkyunkwan University Suwon 16419 Republic of Korea

**Keywords:** broadband detection, fast response, graphdiyne, high responsivity, highly effective heterojunction, MoS_2_

## Abstract

Graphdiyne (GDY), a new 2D material, has recently proven excellent performance in photodetector applications due to its direct bandgap and high mobility. Different from the zero‐gap of graphene, these preeminent properties made GDY emerge as a rising star for solving the bottleneck of graphene‐based inefficient heterojunction. Herein, a highly effective graphdiyne/molybdenum (GDY/MoS_2_) type‐II heterojunction in a charge separation is reported toward a high‐performance photodetector. Characterized by robust electron repulsion of alkyne‐rich skeleton, the GDY based junction facilitates the effective electron–hole pairs separation and transfer. This results in significant suppression of Auger recombination up to six times at the GDY/MoS_2_ interface compared with the pristine materials owing to an ultrafast hot hole transfer from MoS_2_ to GDY. GDY/MoS_2_ device demonstrates notable photovoltaic behavior with a short‐circuit current of −1.3 × 10^−5^ A and a large open‐circuit voltage of 0.23 V under visible irradiation. As a positive‐charge‐attracting magnet, under illumination, alkyne‐rich framework induces positive photogating effect on the neighboring MoS_2_, further enhancing photocurrent. Consequently, the device exhibits broadband detection (453–1064 nm) with a maximum responsivity of 78.5 A W^−1^ and a high speed of 50 µs. Results open up a new promising strategy using GDY toward effective junction for future optoelectronic applications.

## Graphical abstract

Here, for the first time, a highly effective graphdiyne/molybdenum (GDY/MoS_2_) type‐II heterojunction in a charge separation is reported toward a high‐performance photodetector. The device exhibits broadband detection (453–1064 nm) with a maximum responsivity of 78.5 A W^−1^ and a high speed of 50 µs.

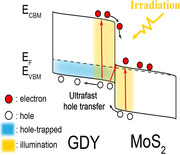

## 1 Introduction

Photodetectors nowadays are attractive with a lot of attention because of wide‐range applications such as thermal images,^[^ ^1^ ^]^ remote control,^[^ ^2^ ^]^ military defenses,^[^ ^1^, ^3^ ^]^ biosensors,^[^ ^1^, ^4^ ^]^ telecommunications,^[^ ^2^, ^5^ ^]^ scientific research.^[^ ^6^ ^]^ Conventional photodetectors are mostly fabricated with inorganic semiconductor materials, such as silicon, InGaAs, HgCdTe, and InSb.^[^ ^7^ ^]^ They still suffer from difficulties such as complex fabrication processes, high cost, lattice mismatch, and strict working conditions, which limit their application for future high‐performance photodetectors.^[^ ^6a^ ^]^ Recently, 2D materials have been considered as a potential candidate for the replacement of traditional materials for photonic applications due to their 1) naturally passivated dangling bonds, which restrict unexpected defect;^[^ ^8^ ^]^ 2) easy construction of heterostructure without lattice mismatch in the presence of weak van der Waals (vdW) forces;^[^ ^8^, ^9^ ^]^ 3) strong light–matter interaction per volume for high performance;^[^ ^10^ ^]^ 4) atomic thickness to lower noise signals for high‐signal‐noise ratio at room temperature;^[^ ^11^ ^]^ 5) low‐cost 2D growth compared with traditional materials.^[^ ^12^ ^]^

So far, there have been studied abundant 2D materials and their photonic properties such as graphene,^[^ ^13^ ^]^ transition metal dichalcogenides (TMDs, e.g., MoS_2_, WSe_2_, and WS_2_),^[^ ^14^ ^]^ and black phosphorus (BP).^[^ ^15^ ^]^ Nevertheless, there are nearly always drawbacks associated with relying solely on a single material. In particular, graphene, one of the most researched 2D materials, possessing ultrahigh mobility (≈10^5^ cm^2^ V^−1^ s^−1^) allow graphene photodetector with ultrafast response time (≈ps) and broadband photodetection up to terahertz.^[^ ^13a^ ^]^ However, the intrinsic low optical absorption coefficient (≈2%) and ultrashort carrier lifetime greatly limits its performance in term of sensitivity, and responsivity.^[^ ^13b^ ^]^ On the contrary, the family of TMDs presents a great potential for high responsivity photodetectors owing to intrinsic strong light–matter interaction and tunable bandgap (1.0–2.5 eV).^[^ ^16^ ^]^ Though, their relatively wide bandgap hinders theirs detection in visible region.^[^ ^16a^ ^]^ Different from graphene and TMDs, bP hold narrow direct bandgap (≈0.3 eV in bulk), accompany moderate carrier mobility (≈5 × 10^3^ cm^2^ V^−1^ s^−1^),^[^ ^17^ ^]^ but it suffers from relatively poor environmental stability.^[^ ^1^ ^]^

Graphdiyne (GDY), a recently discovered carbon allotrope material characterized by its acetylenic bridging benzene rings, has recently emerged as a promising candidate for electronic and optoelectronic applications.^[^ ^18^ ^]^ Different from the zero‐gap of graphene, GDY offers a direct bandgap and exhibits high mobility (2 × 10^5^ cm^2^ v^−1^ s^−1^).^[^ ^18^, ^19^ ^]^ It is considered as a rising star in addressing the challenges posed by graphene's inherent gapless nature and ultrashort carrier lifetime.^[^ ^20^ ^]^ Recently, GDY has been proven with high‐performance photodetectors with a responsivity up to 1260 A W^−1^ in UV region,^[^ ^21^ ^]^ or ultrafast response (≈5 µs) in the visible range.^[^ ^22^ ^]^ However, up to now, there is still no research on 2D vdW heterostructure using GDY for photodetector application. Herein, for the first time, we report a highly effective graphdiyne/molybdenum (GDY/MoS_2_) type‐II heterojunction toward a high‐performance photodetector. Characterized by the robust electron repulsion of alkyne‐rich skeleton,^[^ ^23^ ^]^ the junction exhibits outstanding enhancement in charge separation up to 68.15% compared to graphene/MoS_2_. Transient Absorption spectroscopy (TA) reveals a significant suppression of Auger recombination up to six times at the heterojunction compared with pristine MoS_2_. The GDY/MoS_2_ demonstrates notable photovoltaic behavior with a short‐circuit current of −1.3  × 10^−5^ A and a large open‐circuit voltage of 0.23 V under visible irradiation. Moreover, the alkyne‐rich skeleton acts as positive‐charge‐attracting magnet,^[^ ^21^, ^24^ ^]^ inducing the positive photogating effect on neighboring MoS_2_ layer under illumination. This deeply shifts the Fermi level of MoS_2_ toward the conduction band, further enhancing the output photocurrent. Consequently, the device exhibits broadband detection (453 to 1064 nm) with a maximum responsivity of 78.5 A W^−1^ and a high‐speed of 50 µs. Our results demonstrate the potential of using GDY in photonic applications toward high responsivity, fast response speed, and broadband detection.

## 2 Results and Discussion

### 2.1 GDY Characterization and Device Fabrication

**Figure 1:**
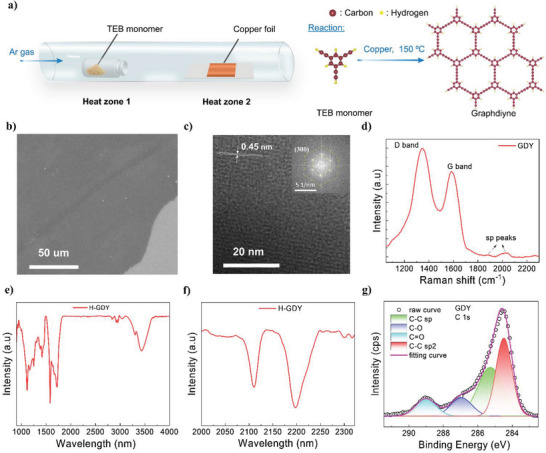
Synthesis procedure and structure characteristics. a) Schematic illustration of GDY synthesis by CVD method. b,c) SEM and TEM images of GDY. Insert: Extracted fast Fourier transform (FFT) pattern. d) Raman spectroscopy of thin film. e,f) full and magnified FTIR spectra. g) C1s XPS spectra of GDY thin film.

The chemical vapor deposition (CVD) schematic illustration and detailed reaction for GDY thin film synthesis are shown in **Figure** **1**a. The 1,3,5‐triethyl‐benzene (TEB) is used as the precursor. Meanwhile, cleaned‐copper foil acted as both the catalyst surface and substrate for the terminal coupling reaction of TEB. The precursor is placed upstream (Heat zone 1) and transported by Ar gas to the copper surface (Heat zone 2). The growth temperature is set at 150 °C under 150 sccm Ar gas. The detailed process is shown in Experiment Section. The scanning electron microscopy (SEM) image of the synthesized film is exhibited in Figure 1b with a large, continuous, and uniform film form. High‐resolution transmission electron microscopy (HRTEM) indicates a high crystallinity structure of the film with a lattice fringe of 0.45 nm (Figure 1c), which is in good agreement with previous reports of GDY.^[^ ^25^ ^]^ The obtained fast Fourier transform (FFT) shows a hexagonal‐shaped dots pattern referring to (300) lattice planes of the GDY, revealing the ABC‐stacking mode of the film (insert Figure 1c).^[^ ^26^ ^]^ TEM results validate the structure of synthesized GDY with the large‐scale crystallinity can be obtained by CVD method. Furthering estimate synthesized structure, Raman and Fourier‐transmission infrared (FTIR) spectroscopies are performed. Raman spectroscopy (Figure 1d) shows 4 distinguished peaks. Peaks observed at 1587 and 1344 cm^−1^ areG and D bands corresponding to the stretching of aromatic rings and disordered/defects in carbon‐framework structure, respectively.^[^ ^27^ ^]^ Two weak peaks at 1888 and 2042 cm^−1^, which are designed for the stretching vibration of conjugated C≡C,^[^ ^28^ ^]^ prove the coupling of terminal alkynes occurred.^[^ ^29^ ^]^ FTIR spectroscopy (Figure 1e) witnesses the strong quenching of ≡ C− H peak at 3301 cm^−1^ compared with the one of TEB (Figure S4, Supporting Information), which strongly verifies the dehydrogenation of TEB precursor during alkyne coupling reaction.^[^ ^30^ ^]^ Also, magnified FTIR spectra at 2000–2350 nm (Figure 1f) show a new peak at 2196 cm^−1^ compared with the TEB corresponding to the presence of adjacent alkyne, which clearly confirms the GDY structure.

To further examine the bonding environment of the prepared film, an X‐ray photoelectron spectroscopy (XPS) measurement is conducted. Figure 1g presents the magnified XPS spectra at C1s peak (284.5 eV). It can be deconvoluted into four sub‐peaks at 284, 285.3, 287, and 289 eV corresponding to the binding energy of C─C sp^2^, C─C sp, C─O, and C═O, respectively. The area ratio of C─C sp^2^ and C─C sp is close to 1:1, which is well‐match with the proposed GDY structure.^[^ ^30^ ^]^ The appearance of C═O and C─O bonds can be assigned from the oxygen physisorption/oxidation of terminal alkyne in the air.^[^ ^28^, ^31^ ^]^ Besides, The UV–vis absorption spectroscopy of GDY is measured, which is shown in Figure S6, Supporting Information. By Tauc‐plot fitting, the calculated optical bandgap of GDY is 2. 72 eV.

**Figure 2:**
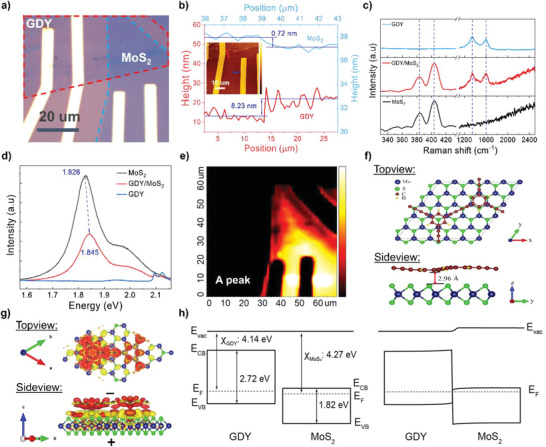
GDY/MoS_2_ heterostructure characterization. a) Optical image of GDY/MoS_2_ photodetector. Red and blue dashed line corresponds to indicate GDY and MoS_2_ area. b) Thickness profiles of individual GDY and MoS_2_. The insert shows the AFM topography image of the GDY/MoS_2_ photodetector. c) Raman of intrinsic GDY, MoS_2_, and GDY/MoS_2_ heterostructure; d,e) PL spectra and mapping of GDY/MoS_2_ photodetector. f) Top and side view of GDY/MoS_2_. The blue, green, brown, and yellow colors show the Mo, S, C, and H atoms, respectively. g) Top‐view and side‐view charge density difference at GDY/MoS_2_ interface. Denotes: The red and yellow isosurfaces correspond to negative and positive charge density after forming junction, respectively. h) Energy band diagram of GDY and MoS_2_ before and after the contacts.

GDY thin film is then transferred on MoS_2_ flakes via the wet transfer method, followed by Cr/Au (5/50 nm) electrode deposition. The detailed processes are shown in Figure S7, Supporting Information. **Figure** **2**a exhibits the optical image of the GDY/MoS_2_ heterostructure photodetector with the GDY region is distinguished by a red‐dash line meanwhile the blue one for MoS_2_. Atomic force microscopy (AFM) characterization (Figure 2b) unveils that the thickness of GDY and MoS_2_ is 8.23 and 0.72 nm, corresponding to multilayers of GDY and a monolayer of MoS_2_.^[^ ^30^, ^32^ ^]^ To investigate the interlayer coupling in GDY/MoS_2_ heterostructure, in situ Raman spectroscopy and mapping are conducted. Raman spectra of GDY, MoS_2,_ and heterostructure are shown in Figure 2c. The observed peaks of pristine GDY and MoS_2_ are match‐well with previous studies.^[^ ^30^, ^32^ ^]^ At heterojunction, the distinct peaks of two component layers are clearly obtained, revealing the good quality of materials after stacking. Raman mapping at distinguish peaks of GDY (D peak) and MoS_2_ (A1g peak) clearly confirms the homogeneous formation over the GDY/MoS_2_ overlapping area (Figure S8, Supporting Information).

### 2.2 High Efficiency Charge Separation and Transfer at GDY/MoS_2_ Junction

To investigate the performance of GDY/MoS_2_ photodetector, an analysis of the charge transfer efficiency at the GDY/MoS_2_ heterostructure is carried out. The photoluminescence (PL) emission and mapping are conducted to probe the charge separation efficiency. With a smaller band gap (1.81 eV) of MoS_2_ accompanied strong light–matter interaction,^[^ ^33^ ^]^ the photogenerated carriers in heterostructure is dominant at MoS_2_. Figure 2d shows the corresponding PL spectra at A (1.826 eV) and B (1.96 eV) exciton peaks of MoS_2_ for GDY, MoS_2,_ and GDY/MoS_2_ heterojunction. Apparently, there is a strong quenching of both A and B exciton peaks at GDY/MoS_2_ heterojunction up 50% compared with pristine MoS_2_. The significant decrease in the PL peak at the heterostructure demonstrates that charge separation occurs efficiently in the heterostructure.^[^ ^2a^ ^]^ In addition, there is an obvious blue shift of the A exciton peak from 1.826 to 1.845 eV, revealing that the photogenerated carriers move from MoS_2_ to GDY. In fact, by PL mapping at A exciton peak (Figure 2e), the efficiency of charge separation at the junction is clearly observed with the strong suppression of the intensity over the overlapping area.

To gain deep insight into the charge separation in GDY/MoS_2_ heterostructure, the simulation approach is conducted (Figure 2f,g). GDY and MoS_2_ monolayer lattice constants are ≈16.33 and ≈3.17 Å, respectively. Figure 2f shows a constructed system in which the 1 × 1 and 5 × 5 supercells of the GDY and MoS_2_ monolayers, respectively, are considered. The large supercell size is used to reduce the amount of forced strain in the heterostructures. The size of the GDY/MoS_2_ heterostructures, as shown in Figure 2f, is 16.01 Å × 13.87 Å in the *x* and *y* plane, and the forced strain is about 2.7% and 5.5%, respectively. The best interlayer spacing in the MoS_2_ monolayer and GDY heterostructure is 2.96 Å. Upon the formation of the heterostructures, the observation of variations in charge density and the distinct charge transfer characteristics provides valuable insights into the change in properties and the electrical structure of the GDY/MoS_2_ heterostructures. Charge transfer practically occurs at the interface with GDY to gain electrons, whereas MoS_2_ loses electrons (Figure 2g), forming the built‐in potential direction from MoS_2_ to GDY. It refers to the alteration of properties brought on by a strong interfacial interaction at GDY/MoS_2_ interface. Additionally, by utilizing the Bader charge quantitative value to assess the charge transfer capability in GDY/MoS_2_ in comparison to graphene/MoS_2_, it is revealed that the electron capture capacity in the GDY exceeds that in graphene/MoS_2_ by up to 68.15%, indicating stronger built‐in potential in GDY/MoS_2_. The favorable charge transfer in GDY/MoS_2_ is well matched with the type II energy band diagram of GDY/MoS_2_ (Figure 2h), which is inferred from UV–vis and ultraviolet photoelectron spectroscopy (UPS) spectroscopy (Figures S6 and S9, Supporting Information).

**Figure 3:**
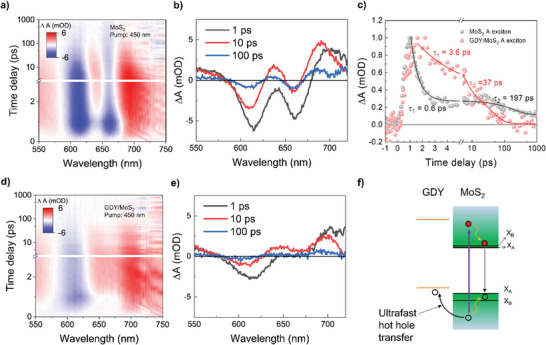
TA characterizations. TA map of a) pristine MoS_2_ and d) GDY/MoS_2_. TA spectra of b) pristine MoS_2_ and e) GDY/MoS_2_ at different time delays of 1 (black), 10 (red), and 100 ps (blue), respectively. c) Normalized TA kinetics of MoS_2_ and GDY/MoS_2_ probed at A exciton energy of 665 nm (1.86 eV). The pump wavelength for TA results was 450 nm (2.76 eV). f) Proposed energy band diagram for efficient‐charge separation in GDY/MoS_2_ heterostructure.

To further clarify charge transfer mechanisms in the GDY/MoS_2_ heterostructure, transient absorption (TA) spectroscopy is conducted. At the pump excitation wavelength of 450 nm, the TA mapping results for the pristine MoS_2_ and GDY/MoS_2_ heterostructure are obtained (**Figure** **3**a,d). Two prominent photobleaching (PB) signals are observed in the A and B excitons of pristine MoS_2_ at 665 and 610 nm, respectively, with decay times of up to 1 ns (Figure 3a). However, in the GDY/MoS_2_ heterostructure, the A exciton signal entirely vanishes, and the intensity of the B exciton signal becomes weaker than the pristine MoS_2_ (Figure 3d), suggesting the ultrafast movement of hot photocarriers out of the MoS_2._ In the plots of TA spectra collected at different time delays of 1, 10, and 100 ps, the reduction of the A and B exciton signals in the MoS_2_ of the heterostructure is more evident (Figures 3b,e). Based on the energy band diagram of GDY/MoS_2_, as shown in Figure 3f, it is suggested that there is an ultrafast hot hole transfer from MoS_2_ to GDY layer. The PB signals originate from the band‐filling of the A and B exciton states by the photoexcited carriers. In MoS_2_, following the generation of B exciton by hole cooling, the A exciton is formed. After ultrafast hole transfer from MoS_2_ to GDY in the heterostructure (Figure 3f), the majority of photoexcited carriers remaining in MoS_2_ of the heterostructure are employed to form the B exciton, making it difficult to form the A exciton due to the lower density of photoexcited carriers in the MoS_2_ of the heterostructure.

We then compare the A exciton TA kinetics of pristine MoS_2_ and GDY/MoS_2_ heterostructure (Figure 3c). The kinetics of A exciton for pristine MoS_2_ rapidly decays at an early time delay right after the A exciton density reaches the maximum intensity and subsequently decays slowly at a later time delay, whereas the reverse tendency is found for MoS_2_ in the heterostructure. Two decay profiles are fitted to the bi‐exponential function, and each time constant is indicated in Figure 3c. For the pristine MoS_2_, the fast decay time of 0.6 ps can be attributed to the Auger recombination due to the high carrier density in the A exciton state, and the slow decay time of 197 ps can be attributed to radiative recombination.^[^ ^34^ ^]^ For the GDY/MoS_2_, the fast decay component becomes slower down to 3.6 ps, while the slower component (37 ps) in the GDY/MoS_2_ becomes faster than the pristine MoS_2_ counterpart. The reduced density of A exciton after ultrafast hole extraction to the GDY valence band slows the Auger recombination by a factor of six and suppresses distinct radiative recombination time, which is consistent with our PL data.

### 2.3 Electrical and Optical Characterization

**Figure 4:**
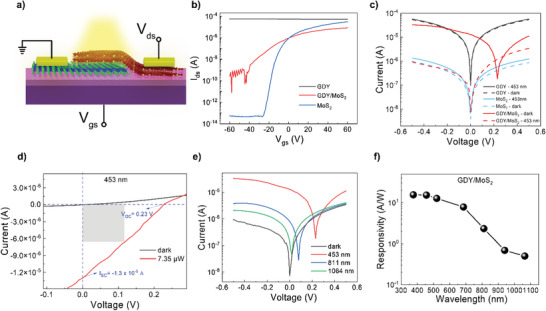
Electrical characterizations. a) Configuration illustration of GDY/MoS_2_ photodetector device under illumination; b) Transfer curves of GDY/MoS_2_ under dark condition. c) *I*_ds_–*V*_ds_ curves of GDY, MoS_2_ and GDY/MoS_2_ under dark and 453 nm laser source. d) Enlarged *I*_ds_–*V*_ds_ curves of GDY/MoS_2_ under dark, and 453 nm laser illumination. e) *I*_ds_–*V*_ds_ curves of GDY/MoS_2_ under 453, 811, and 1064 nm. f) Wavelength‐dependent responsivity ranging from 375 to 1064 nm at *V*_ds_ = −0.5 V and *P*_eff_ ≈ 1 ×  10^−6^ W.

To investigate the electronic properties of GDY/MoS_2_ heterostructure, a back‐gate field effect GDY/MoS_2_ device is fabricated. **Figure** **4**a shows the illustration configuration of the device with the electrode on MoS_2_ grounded, while the one for GDY is biased. The transfer curves of GDY, MoS_2_, and GDY/MoS_2_ heterostructure are shown in Figure 4b. The GDY exhibits good conductivity with p‐type semiconducting characteristics, and the MoS_2_ shows n‐type semiconductor behavior. The field effect mobility (µ_FET_) of GDY and MoS_2_ is inferred following the equation $\mu_{\mathrm{FET}}=\frac{dI}{dV_{g}}.\frac{L}{WC_{g}V_{\mathrm{ds}}}$ where d*I*/d*V*_g_ is the slope of the transfer curve, the applied drain voltage (*V*_ds_), the channel length (*L*), the channel width (*W*), and *C*_g_ as SiO_2_ capacitance.^[^ ^35^ ^]^ The calculated dominant carrier mobility of GDY is 9.044 cm^2^ v^−1^ s^−1^, which is 2.25 times higher than the one of MoS_2_ (Figure S10, Supporting Information). For the GDY/MoS_2_, the transfer curve shows the n‐type behavior with the conductivity significantly suppressed down to around 10^−10^ A at *V*_g =_ −60 V, which is 10^5^ times lower compared to the one of GDY (5 × 10^−5^ A). It is due to the formation of potential barrier height at the GDY/MoS_2_ junction. This suppression in the dark current of GDY/MoS_2_ is greatly important for achieving high sensitivity and responsivity photodetector.^[^ ^36^ ^]^

To explore the photo behavior, the current–voltage (*I*_ds_–*V*_ds_) characteristics of GDY, MoS_2_, and GDY/MoS_2_ are examined under dark and light illumination (Figure 4c). To ensure a fair comparison, a 453 nm (2.73 eV) laser source with a larger photon energy than the energy gap of both GDY (2.72 eV) and MoS_2_ (1.82 eV) is chosen. Results indicate that the pristine GDY and MoS_2_ exhibit no significant responses under 453 nm illumination. By contrast, in the GDY/MoS_2_ heterostructure, a significant increase in the current is obtained with a high photo‐to‐dark current ratio of 10^3^ at zero bias. In addition, there is an upshift under illumination, illustrating the presence of the photovoltaic effect. Figure 4d presents the enlarged *I*_ds_–*V*_ds_ characteristic of the heterostructure. A high short‐circuit current *I*_sc_ of −1.3 × 10^−5^ A and a large open‐circuit voltage of 0.23 V are obtained under 7.35 µW of 453 nm laser illumination. It clearly demonstrates the effective charge separation at GDY/MoS_2_ junction, which is well‐match with the above PL, TA, and simulation results. To further gauge the detection range, the devices undergo additional evaluation using lower photo energy sources, specifically at photon energies below the bandgap values of 1.52 eV (811 nm) and 1.16 eV (1064 nm). As expected, the pristine GDY and MoS_2_ photodetector exhibit a trivial response to light with smaller photon energy than their optical bandgap (Figure S11, Supporting Information). On the other hand, interestingly, the GDY/MoS_2_ heterostructure demonstrates a significant enhancement in current across all examined laser sources from 9.57  ×  10^−7^A under the dark to 2.1  ×  10^−6^ A (1064 nm), 3.8  ×  10^−6^ A (811 nm), and 3.3  ×  10^−5^ A (453 nm) at *V*_ds_ = −0.5 V (Figure 4e), unveiling the broadband detection up to NIR of GDY/MoS_2_ heterostructure. Wavelength‐dependent responsivity of the GDY/MoS_2_ heterostructure (Figure 4f) exhibit a good response ranging from 375–1064 nm at a fixed *V*_ds_ = −0.5 V and light power of ≈1 ×  10^−6^ W, reconfirms the broadband photodetection of GDY/MoS_2_ heterostructure. The significantly higher responsivity in the 375–811 nm range, compared to the 811–1064 nm range, can be attributed to the larger photon energies exceeding the bandgap (*hv* > *E*_g_) of GDY and MoS_2_.

**Figure 5:**
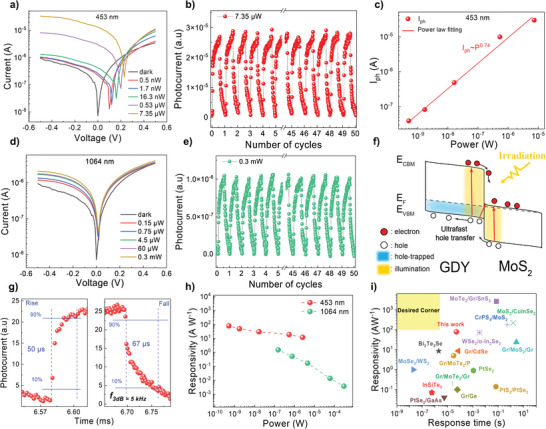
Photoresponse of GDY/MoS_2_ heterostructure photodetector. a–d) *I*_ds_–*V*_ds_ curves of GDY/MoS_2_ heterostructure at different power intensities under 453 and 1064 nm laser illumination, respectively. b‐e) Photo‐switching time‐resolved response of GDY/MoS_2_ photodetector at *V*_ds_ = −0.5 V under 453 and 1064 nm illumination, respectively. c) Power dependent photocurrent *I*_ph_ under 453 nm illumination. f) Energy band diagram of GDY/MoS_2_ under light irradiation. g) Enlarge photo‐switching time‐resolved response of GDY/MoS_2_ photodetector at *V*_ds_ = −0.5 V. h) Power‐dependent responsivity of GDY/MoS_2_ photodetector under 453 and 1064 nm laser illumination. i) Comparison with previous reports based on heterostructure photodetector in terms of responsivity and response time under visible light.

Power intensity plays a vital role in evaluating the performance of the photodetector. Therefore, power intensity‐dependent photoresponse properties of GDY/MoS_2_ are investigated under 453 and 1064 nm laser illumination, as shown in **Figure** **5**a,d. Here, the 453 and 1064 nm are chosen representing photon energies above and below the band gap values, respectively. As expected, with increasing light power, the *I*_ds_–*V*_ds_ curves keep shifting upward in both cases. It is worth noting that the GDY/MoS_2_ device can be detected under a weak signal of 0.5 nW for the visible region and 0.15 µW for the NIR region, exhibiting its ability to detect ultraweak light signals. In addition, the device unveils the long‐term repeatability of the device with almost no degradation up to 50 cycles under laser illumination at *V*_ds_ = −0.5 V (Figure 5b,e). Figure S13, Supporting Information, presents the frequency‐dependent photoresponse ranging from 1 to 100 kHz at fixed *V*_ds_ = −0.5 V. Results exhibit the ultrafast, repeatable, and stable response of the heterostructure up to 100 kHz. The relative balance of [(*I*_max_ − *I*_min_)/*I*_max_] versus time yields a large 3‐dB frequency (*f*_3dB_) of ≈5 kHz. The extracted rising and falling time at *f*_3dB_ are corresponding to 50 and 67 µs, respectively (Figure 5g), which is highly competitive compared with previous reports.^[^ ^1^, ^6^, ^37^ ^]^

Furthermore, the performance of GDY/MoS_2_ photodetector is also studied in terms of photo‐responsivity (*R*) and detectivity (D*). *R* reflects its photosensitivity to incident light following the equation

(1) $$R=\frac{I_{\mathrm{ph}}}{P_{\mathrm{eff}}}$$

where *I*_ph_ is the photocurrent, *P*_eff_ is the effective incident light power. Figure 5h shows the power‐dependent responsivity under 453 nm and 1064 nm irradiation at *V*_ds_ = −0.5 V with highest responsivity in visible and NIR ranges to 78.5 and 1.5 A W^−1^, respectively. The R linearly decreases with the increase of the *P*_eff_ is due to the contracted scattering and recombination.^[^ ^38^ ^]^ On the other hand, D* indicates the capability in detecting weak light signal, which can be expressed as

(2) $$D^{*}=\frac{R\sqrt{A_{\mathrm{device}}\Delta f}}{I_{n}}$$

where *R* is the given responsivity, *A*_device_ is the effective illuminated device area (*A* = 4.22 × 10^−6^ cm^2^), Δ*f* is the given bandwidth, and *I*_n_ is noise current at given bandwidth. Figure S14, Supporting Information, presents the noise current spectrum, in which the noise current density is extracted at our working bandwidth f_3dB_ at Δ*f* = 5 kHz to be ≈1 × 10^−16^ A^2^ Hz^−1^. The calculated D* with the highest values are 7.96  ×  10^8^ and 1.53  ×  10^7^ Jones corresponding to 453 and 1064 nm laser sources, respectively. Figure 5i compares GDY/MoS_2_ photodetector with the previous studies based on 2D heterostructure photodetectors in terms of the responsivity and response time under visible illumination.^[^ ^6^, ^16^, ^39^ ^]^ Significantly, the improvement in both responsivity and speed is obtained in GDY/MoS_2_ photodetector, highlighting the potential of using the GDY/TMD materials approach toward high‐performance future photodetector applications.

### 2.4 Photodetector Mechanism

To gain more insight into the possible operation mechanisms for outstanding performance of GDY/MoS_2,_ power‐dependent photocurrent *I*_ph_ are investigated under 453 and 1064 nm illumination, as shown in Figure 5c and Figure S12, Supporting Information. The extracted *I*_ph_ exhibits a good linear relationship with *P*_eff_ followed by power law fitting of $I_{\mathrm{ph}}\alpha \;P_{\mathrm{inc}}^{\alpha }$,^[^ ^40^ ^]^ with extracted α values are 0.74 and 0.2 corresponding to 453 and 1064 nm, respectively. The obtained α value, being less than one, signifies the existence of photogating effect attributed to the filled‐trap states located within the energy gap of GDY and MoS_2_, which is observed in previous reports.^[^ ^2b^ ^]^ In here, we assume that the intrinsic GDY are not affected by the charge‐trapping induced by MoS_2_ because of the negligible gate dependence in transfer curve of GDY (Figure 4b). Based on results, the energy band diagram of GDY/MoS_2_ can be used to explain the proposed operation mechanism (Figure 5f and Figure S18, Supporting Information). Characterized by strong electron density of alkyne‐rich framework, triple bond in GDY is known as positive‐charge‐attracting magnet, which can act as hole trapping centers underneath of GDY Fermi level.^[^ ^21^, ^24^ ^]^ Under irradiation, the absorbed photons with energies larger than the bandgap (*hv* > *E*_g_) generate electron–hole pairs, then effectively separate at the GDY/MoS_2_ junction, contributing to external circuit. Concurrently, the partially photo‐excited holes trapped underneath the Fermi level of GDY, serve as positive local gate voltage for the adjacent MoS_2_ layer, deeply shifting the Fermi level of MoS_2_ near to the conduction band, further enhancing the output current. In case of photo energies below the bandgap (*hv* < *E*_g_), the photocurrent generates can be explained due to the interlayer transition at small gap (≈0.87 eV) between valence band maximum (VBM) of the GDY and the conduction band minimum (CBM) of MoS_2_. This phenomenon was also observed in other heterostructures.^[^ ^2^, ^41^ ^]^

To clarify this statement, the gate‐dependent photocurrent of in GDY/MoS_2_ have conducted at a fixed *V*_ds_ = −0.5 V under 453 and 1064 nm, which is shown in Figures S16 and S17, Supporting Information. At positive gate bias (*V*_gs_ > 0), the significant enhance of photocurrent in both cases compare with the negative gate bias (*V*_gs_ < 0) due to the upward shifting of MoS_2_ Fermi level. Interestingly, the extracted α displays analogous values at positive *V*_gs_ (0–60 V) of around 0. 7 (453 nm) and 0.2 (1064 nm), (Figures S16b and S17b, Supporting Information). It strongly confirms that the present of positive local gate voltage in GDY/MoS_2_ heterostructure even at *V*_gs_ = 0 V, which is well‐match with the proposed operation mechanism.

## 3 Conclusion

Here, for the first time, the GDY/MoS_2_ heterostructure is fabricated for photodetector application. The outstanding performance of GDY/MoS_2_ photodetector can be assigned due to: 1) The highly effective charge separation and transfer at GDY/MoS_2_ junction; and 2) The present of a local positive gate voltage induced by the strong electron repulsion of the triple bond in GDY on the neighboring MoS_2_ layer under irradiation. The efficient charge transfer behavior at the GDY/MoS_2_ interface is studied by PL and TA. Results reveal the strong suppression of Auger recombination at the GDY/MoS_2_ interface is up to six times compared with pristine MoS_2_. Simulation results prove that the effective charge transfer at GDY/MoS_2_ interface is 68.15% better than that at the graphene/MoS_2_ interface. The device demonstrates a broadband detection ranging from 375 to 1064 nm with a high responsivity in broadband up to 78.5 (453 nm) and 1.5 A W^−1^ (1064 nm). Furthermore, a fast‐rising and recovery time of GDY/MoS_2_ are obtained up to 50 and 67 µs, respectively. Our results prove the effectiveness of GDY/MoS_2_ heterojunction toward high‐performance photodetector and open up the applicable potential using GDY toward high‐performance optoelectronic applications.

## 4 Experimental Section

### Chemicals

1,3,5‐tribromobenzene (98%), tetrakis(triphenylphosphine)palladium (0) (99%), copper(I) iodide (≥99.5%), trimethylsilyl acetylene (≥98.0%), sodium hydroxide (≥99.0%), methyl alcohol (HPLC, ≥99.9%), hexane (99%), poly (methyl methacrylate), sodium molybdate (≥98.0%), optiPrep density gradient medium were purchased from Sigma‐Aldrich Inc. and used without purifications. The details synthesis processes are in Supporting Information.

### Synthesis of TEB and GDY film

TEB was synthesized following the previous, as shown in Supporting Information. GDY was synthesized on copper foil by CVD using TEB as the precursor and cleaned‐copper foil as the substrate. The precursor was placed upstream at heat zone 1. Meanwhile, the substrate was at heat zone 2. First, copper was annealed at 890 °C under 350 sccm argon gas for 10 min, followed by natural cooling to room temperature. The growth processes start by simultaneously ramping heat zone 1 and 2 to 60 and 150 °C, respectively, at 150 sccm Ar gas. The typical growth took 4 h, followed by natural cooling down. The GDY‐on copper foil was then loaded out and coated with polymethyl methacrylate (PMMA) as a protective layer for further steps.

### MoS_2_ CVD Growth

The MoS_2_ flakes were obtained using the standard CVD system using Na_2_MoO_4_ solution (0.024 g mL^−1^) as a Mo precursor. An OptiPrep density gradient (Opti) adhesive solution was added to Na_2_MoO_4_ in a ratio Na_2_MoO_4_/Opti/DI = 0.5/0.5/3. For the growth of MoS_2_ monolayer, the precursor‐coated substrates were placed at the center of the furnace, and the S powder was placed in the upstream region. The sample was ramped to 800 °C for 25 min under 100 sccm N_2_ gas. The growth starts at 800 °C for 5 min to obtain MoS_2_ monolayer, followed by cooling down to room temperature.

### Device Fabrication

For device fabrication, MoS_2_ flakes and GDY were sequentially transferred onto a SiO_2_/Si substrate (300 nm‐thick SiO_2_) using a wet transfer approach. The PMMA solution was spin‐coated on the substrate at 3000 rpm for 60 s, followed by banking at 130 °C for 3 min. The electrodes were defined by electron‐beam lithography, followed by Cr/Au (5/50 nm) deposition using electron‐beam evaporation. Finally, the residue PMMA was removed by a lift‐off process using acetone.

### Structure Characterization

Nuclear magnetic resonance (NMR) spectra were recorded by 500 MHz NMR (Bruker). SEM images were obtained by JEOL 7500F FESEM. The HRTEM images were obtained using JEM‐ARM200CF, JEOL system with an accelerating voltage of 200 kV. Raman and Photoluminescence measurements were obtained using an NTE‐GRA spectra instrument (NT‐MDT) equipped with a 532 nm laser at room temperature. For FTIR, a Vertex 70 (Bruker) instrument was used in transmission mode. The absorption measurement was measured by UV−vis/NIR spectrometer (Jasco, V770). XPS measurement is conducted by Thermo VG Microtech ESCA 2000 with a monochromatic Al‐Kα X‐ray source at 100 W. The binding energy scale was calibrated by referencing C 1s to 284.5 eV. UPS was performed under high vacuum conditions by ESCALAB250 (Thermo‐Scientific, U.K.) instrument. AFM measurements were carried out using DFM mode of AFM SPA400, SEIKO system.

### Electrical and optoelectronic characterizations

The electrical and optoelectronic behavior were performed under high‐vacuum conditions (≈10^−6^ torr) at room temperature using semiconductor analyzer Keithley 4200 system under dark, 375, 453, 520, 685, 811, 940, and 1064 nm lasers (RGB photonics). For time‐dependent photocurrent response, the oscilloscope (DSO‐X 2002A, Agilent Technologies) and a 453 nm laser is used with varying the laser on/off frequency using a function generator (DS345, Stanford research). For noise current measurement, the homemade low frequency noise was used, which the system sets up is shown in Figure S15, Supporting Information. The system consisted of a DC voltage source, a current preamplifier (SR 570), and a data‐acquisition card (DAQ‐4431, National Instruments). The system was controlled by LabView program (National Instruments). The noise density spectra were measured at −0.5 V under dark conditions, high vacuum condition (≈1 × 10^−7^ torr).

### TA Measurement

Ti:sapphire amplifier (Legend Elite, Coherent) was used to generate a 790 nm 1‐kHz laser and divided into two beams. One was used for the probe beam by generating the white light continuum (500–800 nm) with a nonlinear crystal. Another was used for the pump beam by sending it to an optical parametric amplifier (TOPAS Prime, Light Conversion) to generate a 450 nm wavelength light with an energy per pulse of 0.1 nJ. The pulse duration time was ≈30 fs. The confocal TA microscope system (HELIOS, Ultrafast Systems) with two 50 × objective lenses was used for micro‐TA measurement, and the data were acquired with the transmittance mode.

### Computational Details

The Vienna ab initio simulation package's spin density functional theory was used for all the calculations. For electron–electron exchange correlations, the projector augmented wave method was utilized, whereas the generalized gradient approximation with the Perdew–Burke–Ernzerhof parametrization scheme was utilized for electron–ion interactions. The cutoff energy for the plane‐wave expansion of electronic eigenfunctions in the pure 2D GDY, graphene, the MoS_2_ monolayer, and the heterostructure GDY/MoS_2_ and graphene/MoS_2_ (for comparison) was used to be 450 eV. The van der Waals energy correction was accounted for in nanocomposite by utilizing Grimme's semi‐empirical correction scheme. The force criterion for structural relaxation was 0.001 eV Å^−1^. Along the direction perpendicular to the 2D nanosheet, 15 Å of additional vacuum separation was added to prevent interference and periodic collisions between neighboring layers. For Brillouin zone integration, Γ‐point sample of 4 ×  4 ×  1 *k*‐points of the nanocomposite was utilized. Bader charge analysis was utilized to quantify the amount of charge transferred from MoS_2_ to GDY (or graphene). The charge density difference (Δρ) was expressed as: $\Delta \rho =\rho_{{\mathrm{MoS}}_{2}/\mathrm{GDY}}-\rho_{{\mathrm{MoS}}_{2}}-\rho_{\mathrm{GDY}}$, where $\rho_{{\mathrm{MoS}}_{2}/\mathrm{GDY}}$, $\rho_{MoS_{2}}$, ρ_GDY_ represent the charge density of GDY/MoS_2_, virgin MoS_2_, and GDY, respectively, obtained from similar atomic locations and unit cells of the same size.

## Conflict of Interest

The authors declare no conflict of interest.

## Author Contributions

D.P.D. performed experiments and wrote the manuscript. C.H. set up, analyzed, and wrote TA results. H.P. and B.Q.V. conducted the simulations. S.S. gave the comments during the project and revised the manuscript. D.D.V. synthesized MoS_2._ T.L.P. supported conceptualization and electronic characterizations. K.M.T. and S.H. supported TEB synthesis. W.J.Y. supported D.D.V. and T.L.P. in synthesis. B.A. and S.C.L. supported noise spectrum measurement, S.G.K. provided a hardware system for computational simulations. J.H.K. participated in writing TA results. H.L., as a project manager, discussed issues and gave comments, and revised the manuscript.

## Supporting information

Supporting Information

## Data Availability

The data that support the findings of this study are available from the corresponding author upon reasonable request.
