# Supplementary material for: Highly Efficient Van Der Waals Heterojunction on Graphdiyne toward the High‐Performance Photodetector
Source: Adv Sci (Weinh). 2023 Jul 9;10(25):2300925. doi: 10.1002/advs.202300925 (PMC10477878; doi:10.1002/advs.202300925)
Supplement: Supplementary file 1 — Supporting Information [file ADVS-10-2300925-s001.pdf]

ADVANCED SCIENCE
Open Access

Supporting Information

for *Adv. Sci.*, DOI 10.1002/advs.202300925

Highly Efficient Van Der Waals Heterojunction on Graphdiyne toward the High-Performance Photodetector

Dinh Phuc Do, Chengyun Hong, Viet Q Bui, Thi Hue Pham, Sohyeon Seo, Van Dam Do, Thanh Luan Phan, Kim My Tran, Surajit Haldar, Byung-wook Ahn, Seong Chu Lim, Woo Jong Yu, Seong-Gon Kim, Ji-Hee Kim and Hyoyoung Lee**

SUPPORTING INFORMATION

Highly Efficient Van Der Waals Heterojunction on Graphdiyne toward the High-performance Photodetector

Dinh Phuc Do, Chengyun Hong, Viet Q.Bui, Thi Hue Pham, Sohyeon Seo, Van Dam Do, Thanh Luan Phan, Kim My Tran, Surajit Haldar, Byung-wook Ahn, Seong Chu Lim, Woo Jong Yu, Seong-Gon Kim, Ji-Hee Kim, and Hyoyoung Lee**

D.P. Do, Dr. S. Seo, K.M. Tran, Dr. S. Haldar, Prof. H. Lee

Department of Chemistry

Sungkyunkwan University

Suwon, 16419, Republic of Korea

Email: hyoyoung@skku.edu

C. Hong, B. Ahn, Prof. S.C. Lim, Prof. JH. Kim

Department of Energy Science

Sungkyunkwan University

Suwon, 16419, Republic of Korea.

Email: **kimj@skku.edu**

T. H. Pham, Dr. V. Q.Bui

Advanced Institute of Science and Technology

The University of Danang

41 Le Duan, Danang, 92026, Vietnam.

Dr. S. Seo, Prof. H. Lee

Creative Research Institute

Sungkyunkwan University

Suwon, 16419, Republic of Korea.

V.D. Do, Dr. T.L. Phan, Prof. W.J. Yu

Department of Electrical and Computer Engineering

Sungkyunkwan University

Suwon, 16419, Republic of Korea

Prof. S.G. Kim

Department of Physics and Astronomy and Center for Computational Sciences

Mississippi State University

Mississippi State, MS, 39762, USA

Prof. H. Lee

Department of Biophysics

Sungkyunkwan University

Suwon, 16419, Republic of Korea

KEYWORDS: Graphdiyne (GDY), Molydenum (MoS_2), highly effective heterojunction, high responsivity, fast response, broadband detection.

Table S1: Comparison table of the performance of GDY/MoS_2 photodetector with previous graphene (Gr) and TMDs-based heterostructure.

Device	Range (nm)	V_{ds} (V)	Responsivity (A/W)	Detectivity (J)	Response time	On/off ratio	Ref
**GDY/MoS_2**	**453 - 1064**	**- 0. 5**	**78.5 (453 nm)**<br>**1.5 (1064 nm)**	**9×10^{10} (453 nm)**<br>**1.8×10^{9} (1064 nm)**	**50** μs, **67** μs	**10^3**	**This work**
$MoSe_2/WS_2$	780	2	1	N/A	0.02 μs, 0.03 μs	10^4	[1]
Gr/MoS_2/Gr	405 - 904	3	23.95 (532 nm)<br>23.5×10^{-3} (904 nm)	N/A	2.7 s	N/A	[2]
Gr/$MoTe_2$/P	520 - 1400	0	5 (520 nm)<br>~2 (1064 nm)	3×10^{12} (520 nm)	30 μs	N/A	[3]
$InSiTe_3$	365 - 1310	11	7.1×10^{-2} (365 nm)<br>$\sim 2 \times 10^{-3}$ (1310 nm)	7.59×10^{9} (365 nm)<br>$\sim 2 \times 10^{8}$ (1310 nm)	0.576 μs	N/A	[4]
Gr/$MoTe_2$/Gr	473 - 1064	0	0.205 (475 nm)<br>0.11 (1064 nm)	N/A	24 μs, 46 μs	N/A	[5]
Bi_2Te_2Se	365 - 980	5	8.53 (365 nm)<br>2.74 (980 nm)	N/A	2 μs	2.2×10^3	[6]
$MoTe_2$/Gr/SnS_2	405 - 1550	1	10^2 - 10^3 (All range)	1.1×10^{13}	17.6 ms, 72.3 ms	N/A	[7]
$CrPS_4/MoS_2$	532 - 1450	2	~ 175 (532 nm)<br>7.69×10^{-3} (1095 nm)	3.21×10^{9} (532nm)<br>1.35×10^{9} 1095 nm)	0.5 s	N/A	[8]
$PtS_2/PtSe_2$	405 - 2200	0	0.14 (405 nm)<br>0.06 (1064 nm)	N/A	66 ms, 5 ms	N/A	[9]
WSe_2/α-In_2Se_3	400 - 1100	- 3	72.4 (520 nm)<br>2.21 (980 nm)	1.76×10^{12} (520 nm)<br>9.52×10^{12} (980 nm)	3.3 ms, 5.7 ms	10×10^3	[10]
$PtSe_2$/GaAs	254 - 1200	0	18×10^{-3} (~453 nm)<br>262×10^{-3} (808nm)	2.5×10^{12} (808nm)	5.5 μs, 6.5 μs	3×10^4	[11]
Gr/Ge	450 - 1627	0.5	< 0.1 (450 nm)<br>0.75 (~1400 nm)	2.53×10^{9}	60 μs, 60 μs	1.6	[12]

Gr/CdSe	325 - 633	N/A	8.7 (633 nm)	N/A	70 µs, 137 µs	N/A	[13]
MoS2/$CuInSe_2$	355 - 1064	1	220.4 (355 nm)<br>74.8 (1064 nm)	2×10^{12} (355nm)<br>7.1×10^{11} (1064nm)	1.5 s, 1.2 s	> 50	[14]
$PtSe_2$ monolayer	632 - 1470	0.1	0.9 (632 nm)<br>0.15 (1470 nm)	N/A	1.1 ms, 1.2 ms	N/A	[15]

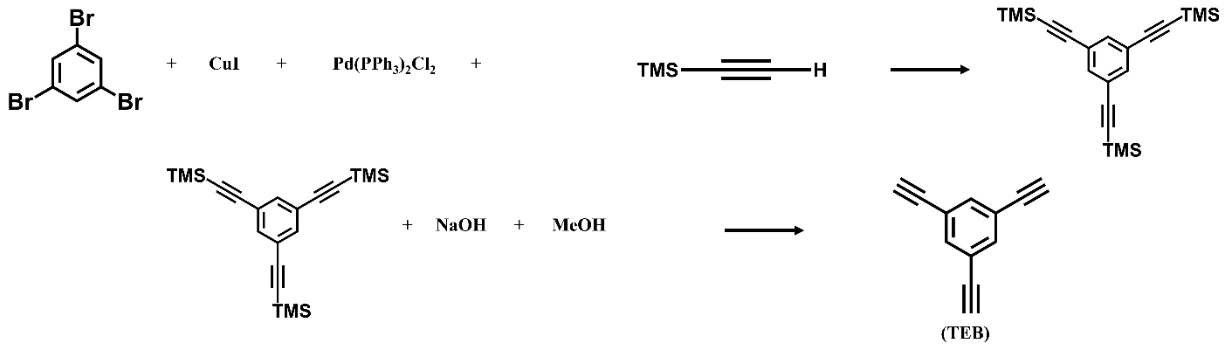

Figure S1. Synthesis scheme of 1,3,5-triethynyl benzene (TEB)

Synthesis of 1,3,5-tris(trimethylsilyl-ethynyl) benzene (TEB-TMS)

1,3,5-Tribromobenzene (250mg, 0.79mmol) is dissolved in dry triethylamine (0.452 ml) were added Pd(PPh3)4 (35.1 mg, 0.05 mmol) and CuI (9.55mg, 0.05 mmol) under inert Ar atmosphere. After the mixture completely dissolves, slowly add trimethylsilyl acetylene (0.452 ml, 3.16 mmol) under the refluxed condition at 60^0C for 16h. Once the reaction is complete, the solvent is evaporated, followed by silica gel column chromatography (n-hexane) to give the 1,3,5-tris(trimethylsilyl-ethynyl) benzene (TEB-TMS) (283 mg, 98%) as pale-yellow solid.

^{1}H NMR ($CDCl_3$, 500 MHz, ppm): δ 7.42 (s, 2.75), 0.19(s, 27H).

^{13}C NMR ($CDCl_3$, 500 MHz, ppm): δ 134.7, 123.69, 102.86, 95.69, 53.65, 0.23.

Synthesis of 1,3,5-Triethynylbenzne (TEB)

This product is then dissolved in CH_2Cl_2 (2.46 mL), followed by adding a mixture of methanol/ sodium hydroxide (2.46 mL, 0.186mg). Then, the mixture is stirred under Ar gas at room temperature for 12h. Once the reaction is complete, the solvent evaporates. Finally, the crude is worked-up by CH_2Cl_2, water, and brine, followed by dry over anhydrous Na_2SO_4 to give the 1,3,5-triethynylbenzenze as the white color (111 mg, 96%).

^{1}H NMR ($CDCl_3$, 500 MHz, ppm): δ 7.57 (s, 3H), 3.1 (s, 3H).

^{13}C NMR ($CDCl_3$, 500 MHz, ppm): δ 135.86, 123.12, 81.82, 78.89.

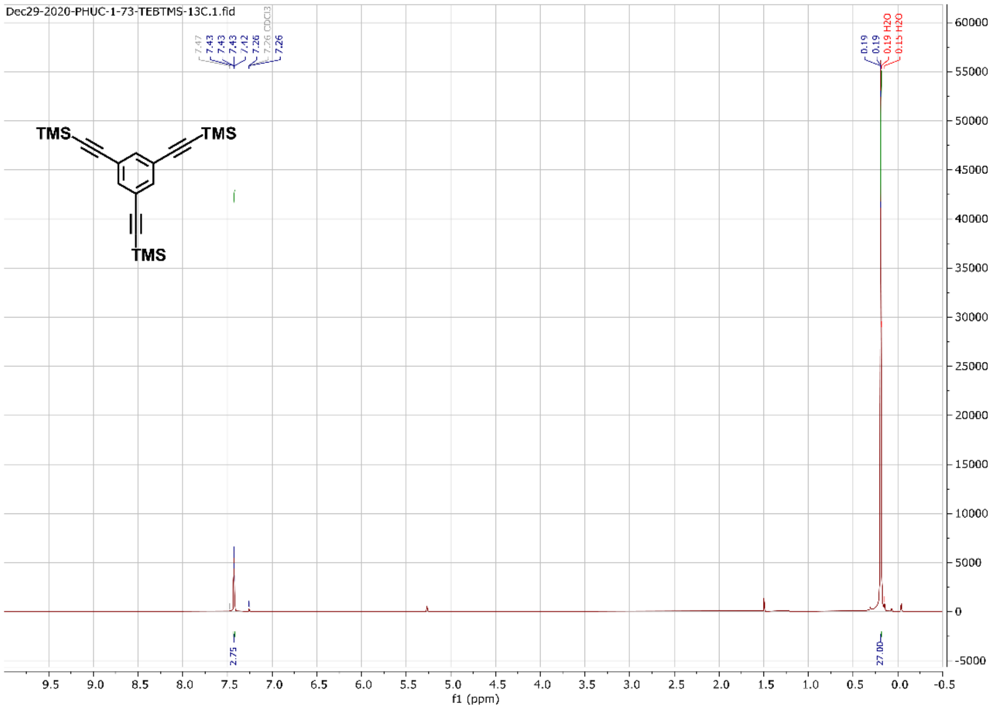

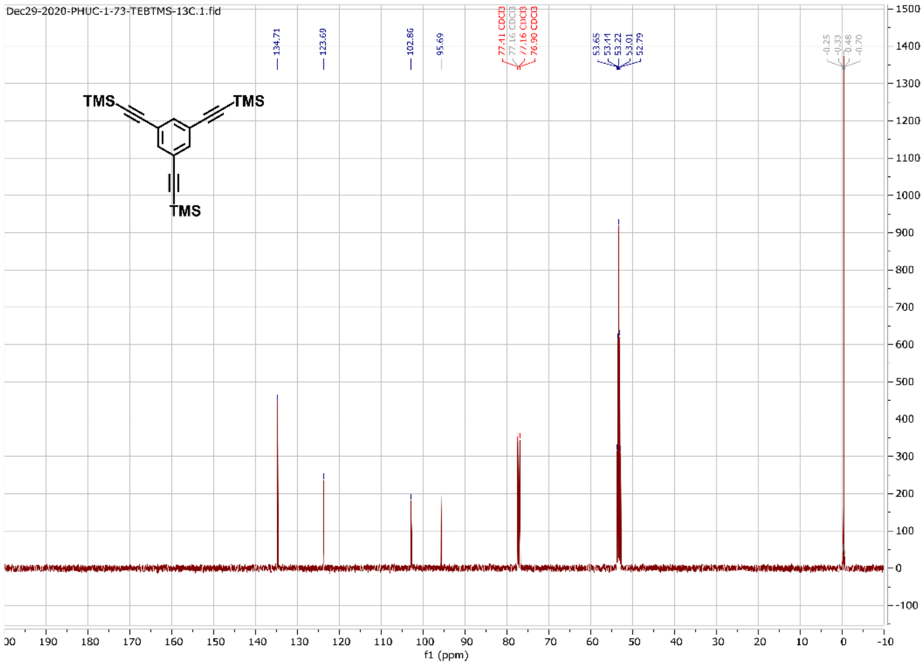

Figure S2. ^{1}H NMR and ^{13}C NMR of TEB-TMS.

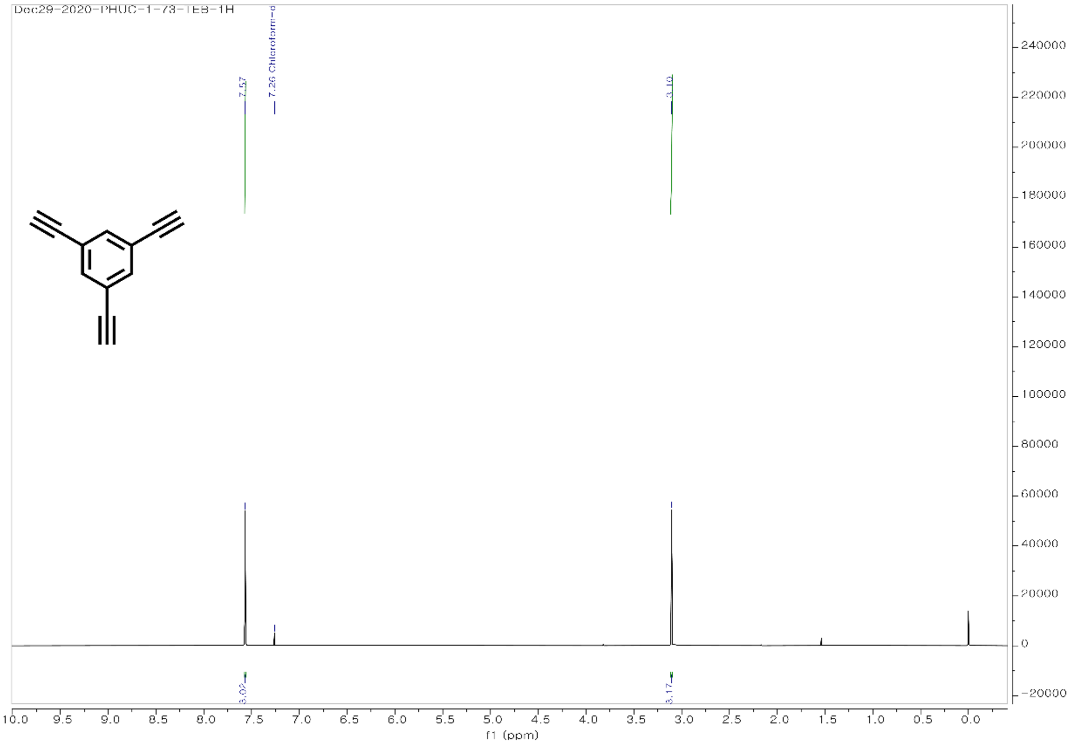

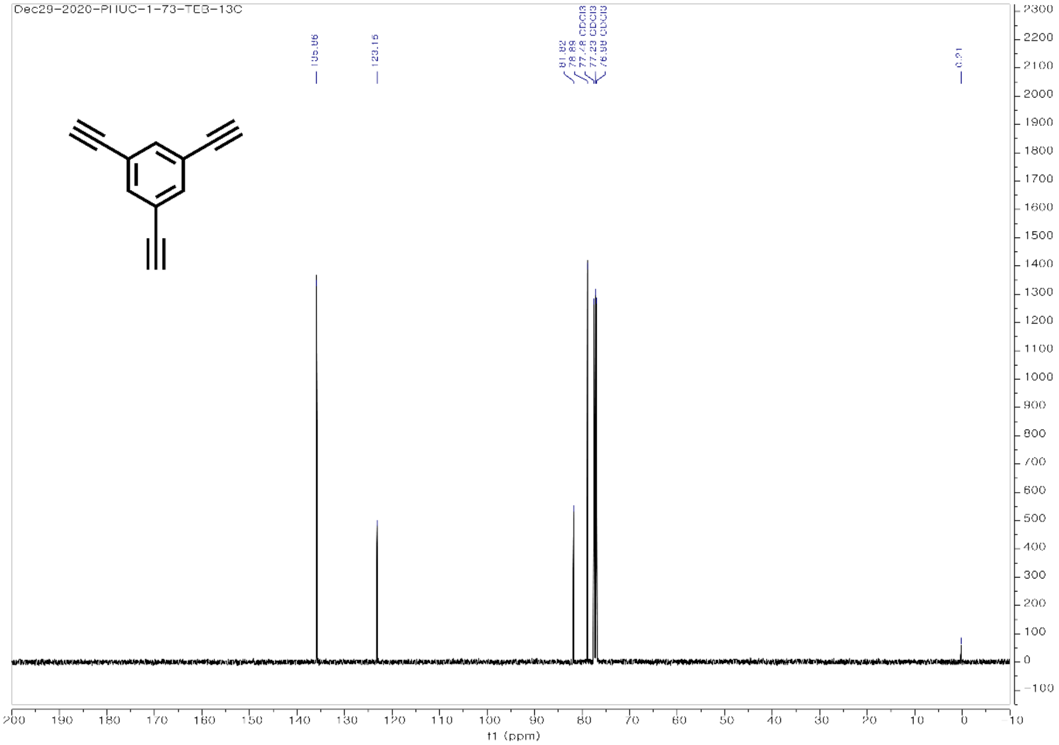

Figure S3. ^{1}H NMR and ^{13}C NMR of TEB

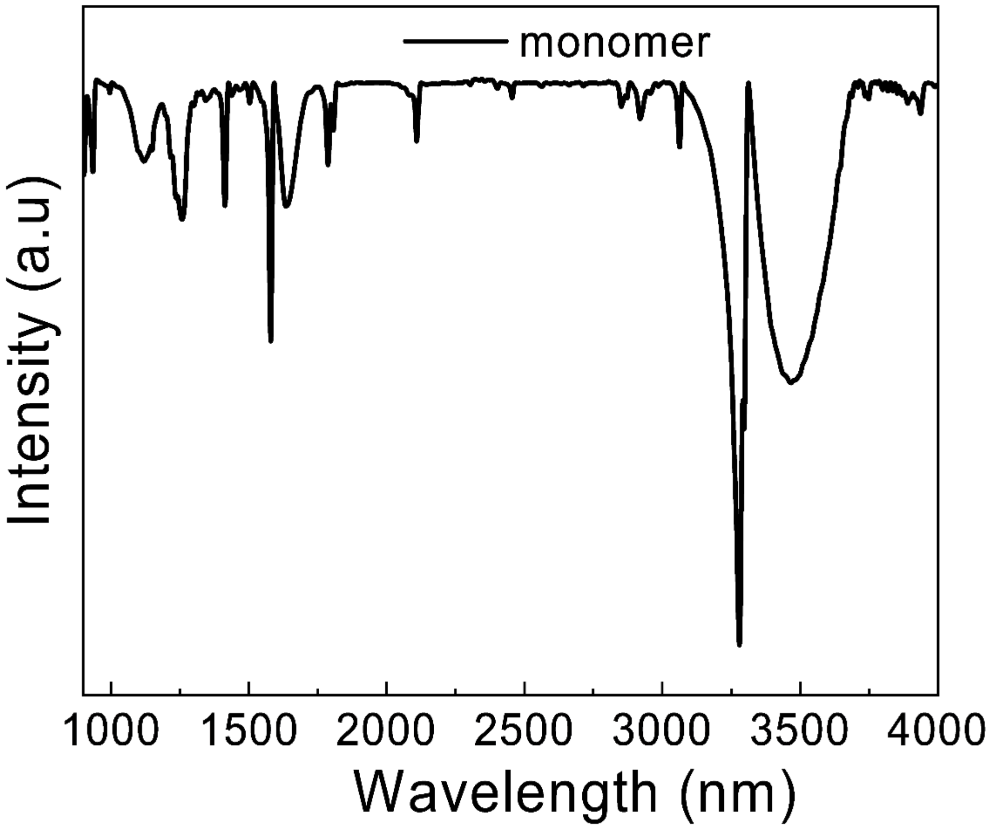

Figure S4. FT-IR of TEB monomer.

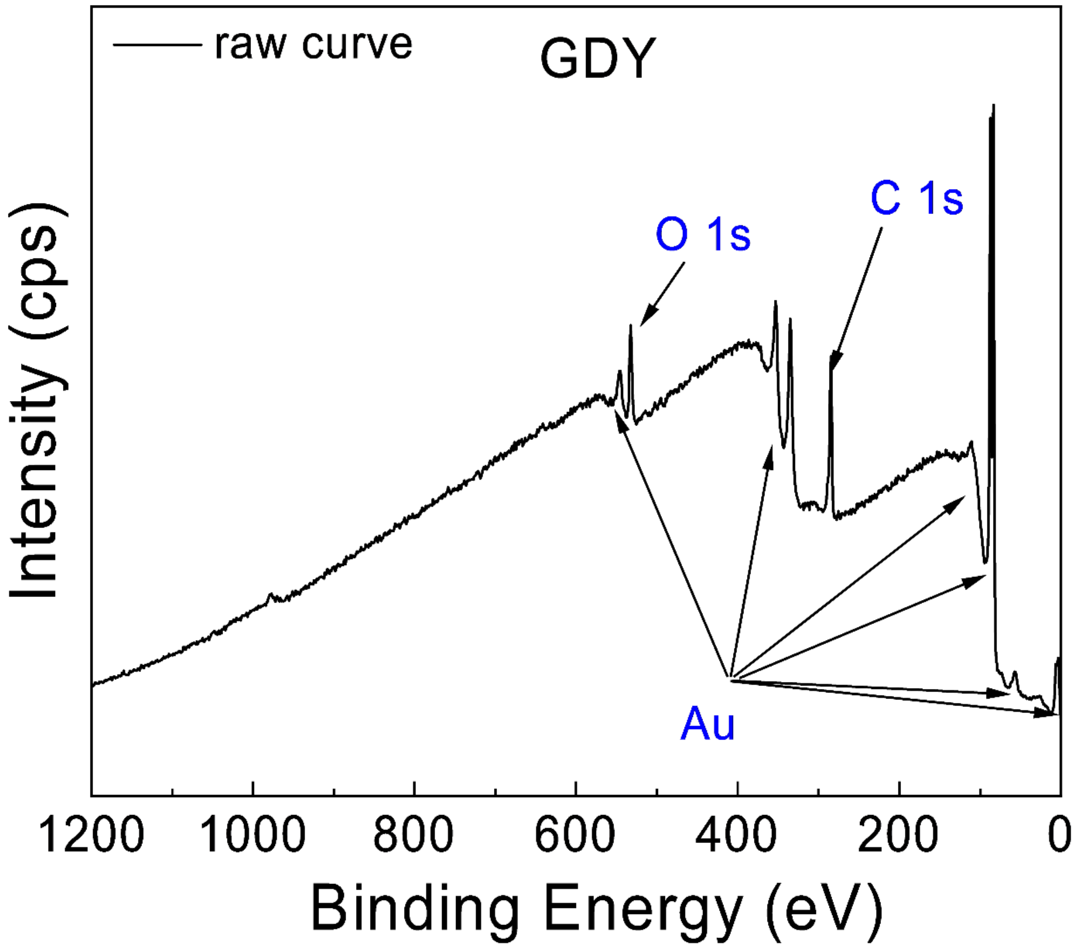

Figure S5. Full XPS spectra of H-GDY on 100nm Au substrate.

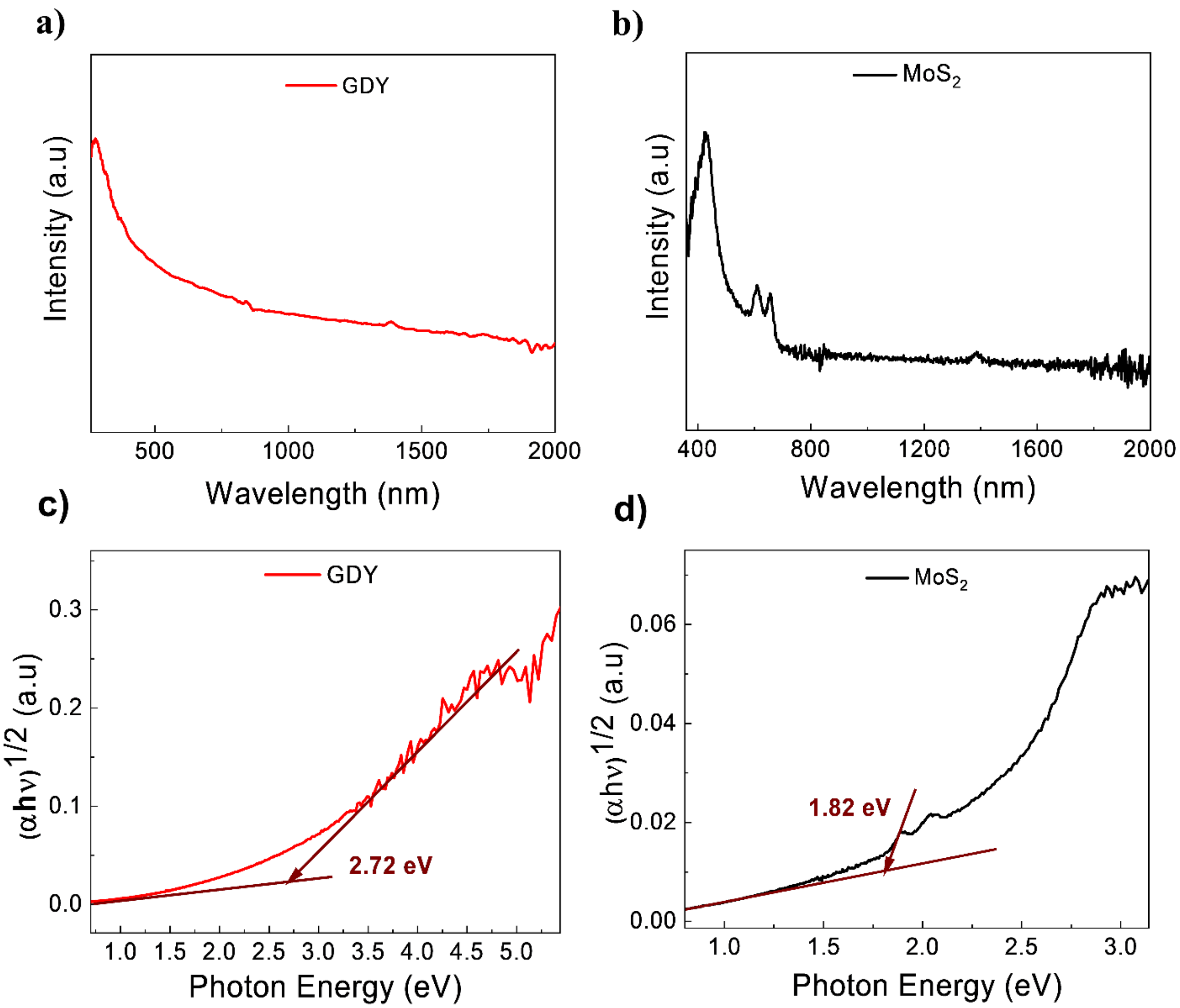

Figure S6. Uv-vis absorption spectra and extracted optical bandgap by Tauc-plot function of GDY.

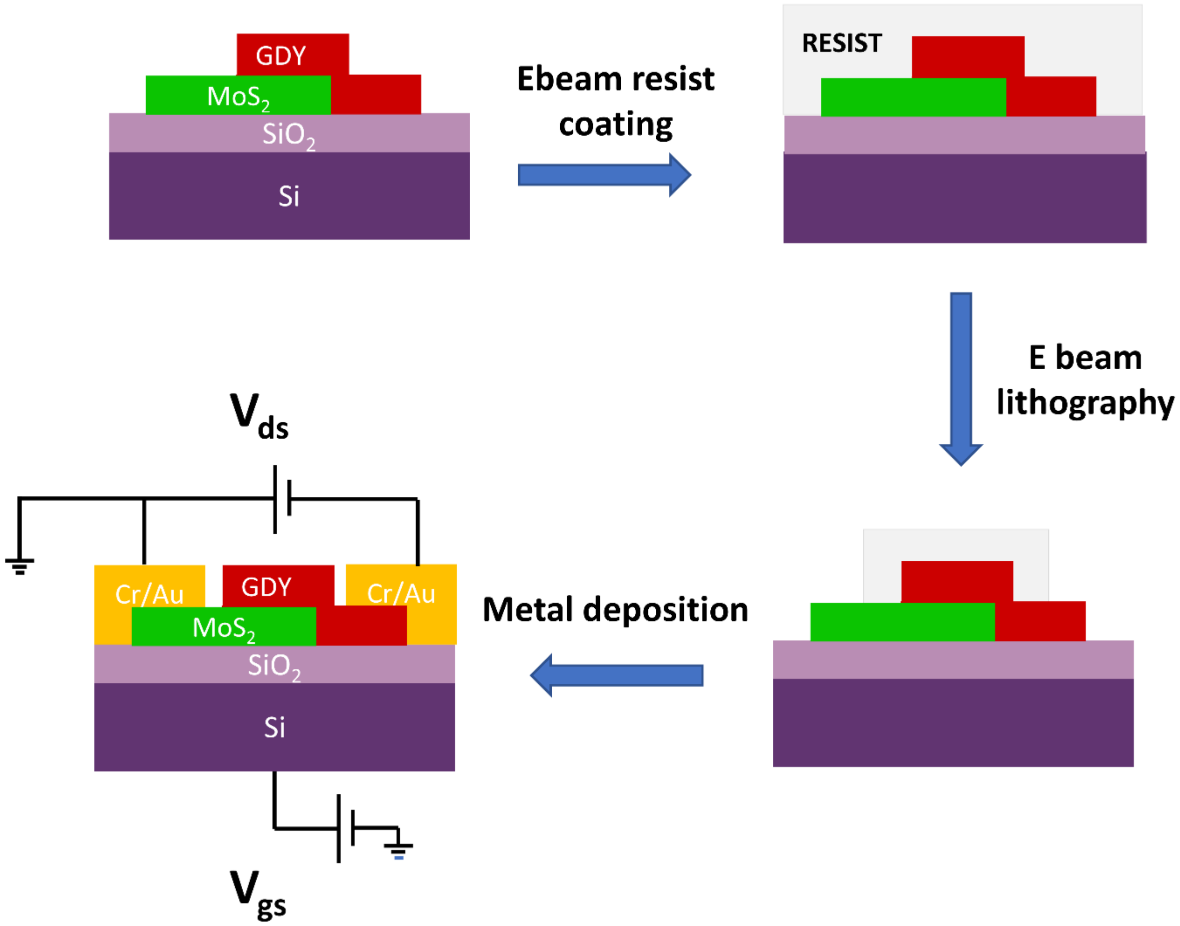

Figure S7. GDY/MoS_2 device fabrication process.

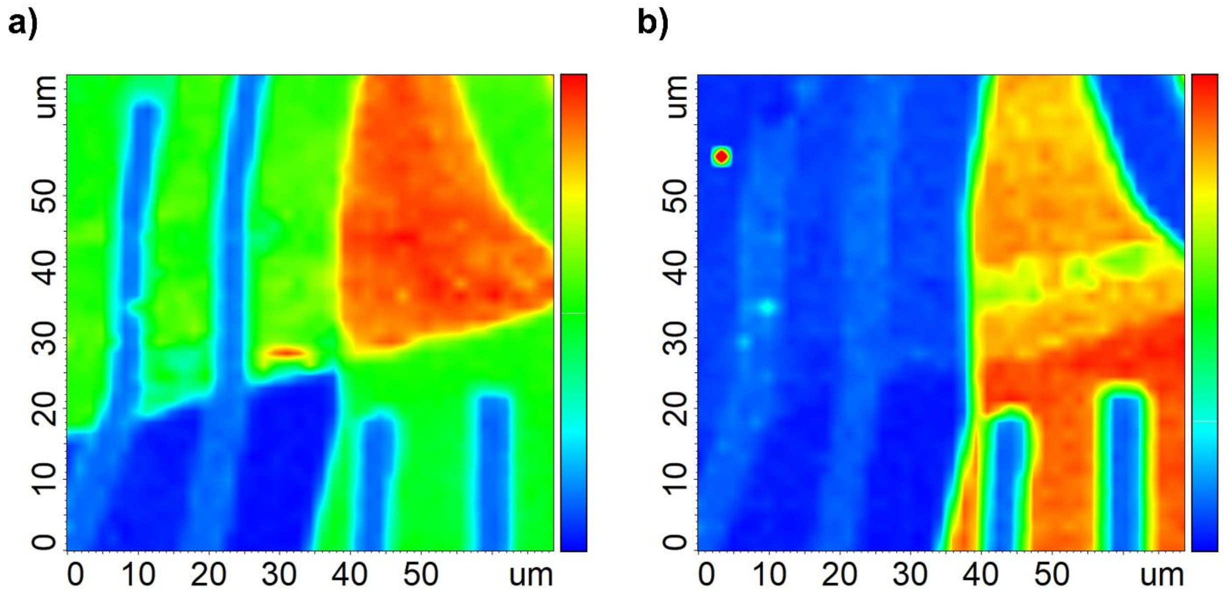

Figure S8. Raman mapping of GDY/MoS_2 photodetector at a) D peak and b) A_{1g} peak.

At heterojunction, the intensity of the D peak increases significantly compared with pristine GDY, which is induced by the disordered carbon-framework system since interlayer interaction with MoS_2, which was reported in other 2D heterostructures. The similar result obtains at A_{1g} peaks for MoS_2 reaffirms the successful construction of GDY/MoS_2 heterojunction.

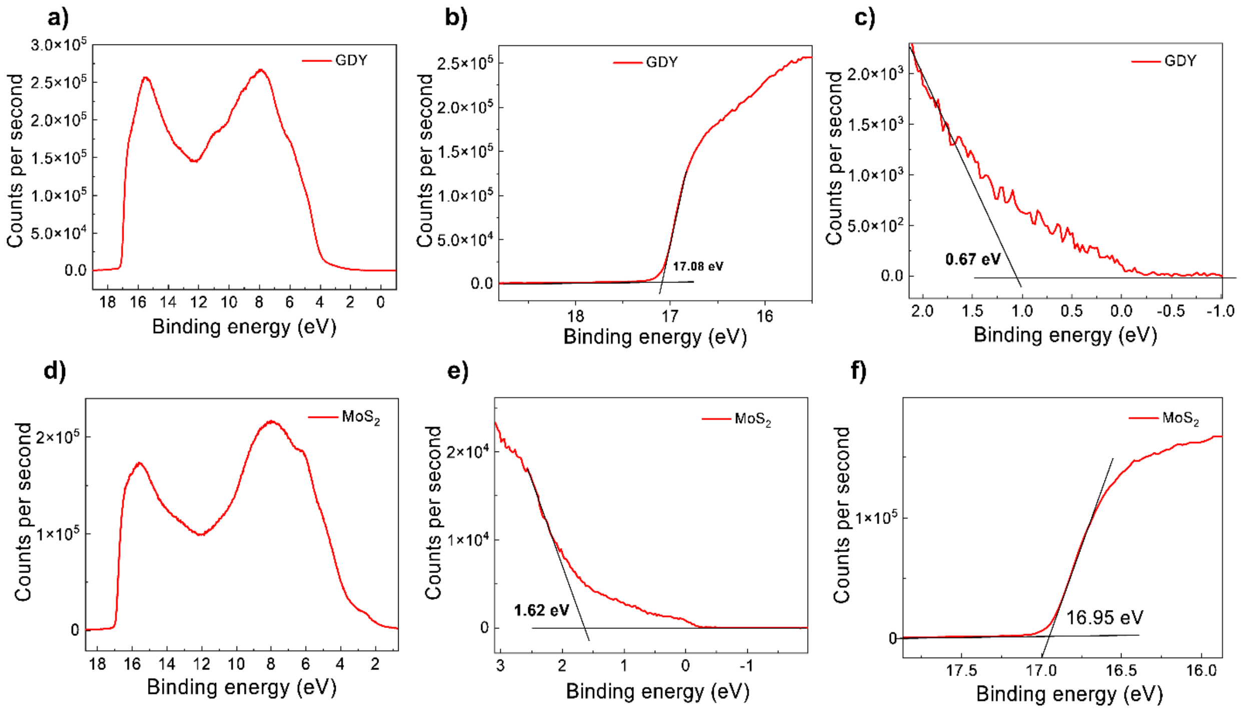

Figure S9. Ultraviolet photoelectron spectroscopy (UPS) of a) GDY and c) MoS_2. b), e) The extracted work function (ϕ) and c), f) the energy difference between the fermi level (E_f) and valence band maximum (E_{VBM}) of GDY and MoS_2, respectively.

To easier explain the carrier movement behavior at the junction, ultraviolet spectra (UPS) are used to calculate the relative position of Fermi energy (E_f) and valence band maximum (VBM) of GDY and MoS_2, ultraviolet spectra (UPS) are conducted. GDY and MoS_2 is transferred on 100nm Au-coated Si substrate**.** The work function (ϕ) can be inferred using the equation ϕ= hv- E_{onset}, where hv is the incident photon energy (21.22 eV) [15] and E_{onset} is the onset level related to the secondary electron.[15]. The calculated ϕ values of GDY and MoS_2 are 4.14 and 4.27 eV, respectively. Additionally, the relative valence band maximum (VBM) position compared with Fermi level (E_f) can be inferred from the cutoff of lowest binding energy, which is 0.67 eV and 1.62 eV for GDY and MoS_2, respectively (Figure S9, Supporting

Information). Combined with the optical bandgap of GDY and MoS_2 is 2.72 eV and 1.82 eV, respectively (Figure S6), the energy band alignment diagram of individual GDY and MoS_2 and after contact is shown in **Figure 2**h.

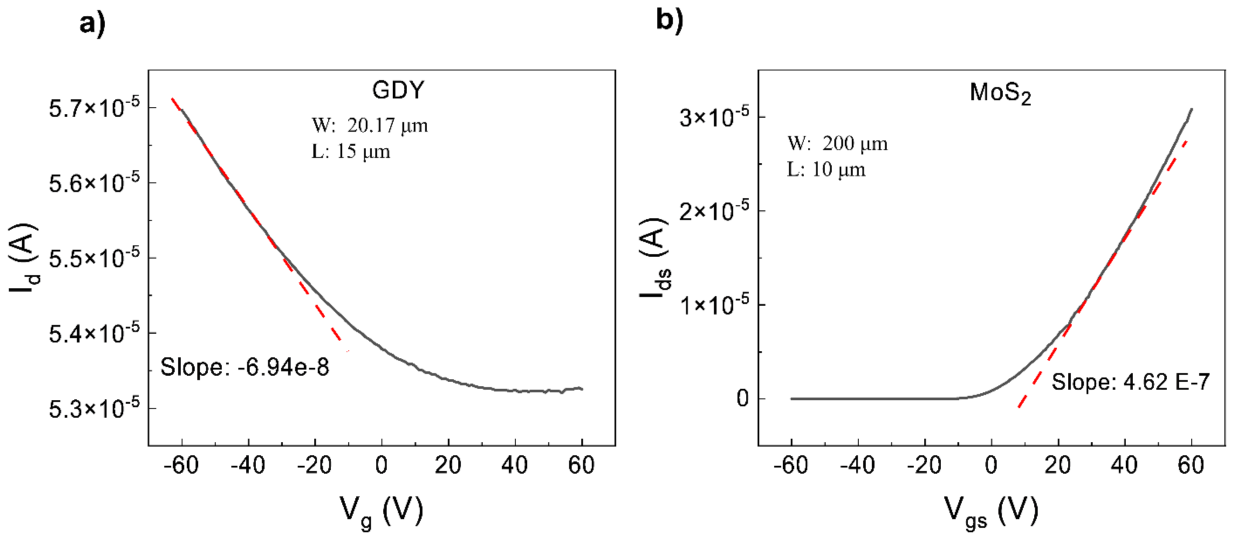

Figure S10. Transfer curve of a) GDY and b) MoS_2.

The field effect mobility (μ_{FET}) of GDY and MoS_2 can be extracted using the equation $\mu = \frac{dI}{dV_g} \cdot \frac{L}{WC_gV_{ds}}$, where dI/dV_g is the slope of the transfer curve, the applied drain voltage (V_{ds}), the channel length (L), the channel width (W), and Cg as SiO_2 capacitance[16].

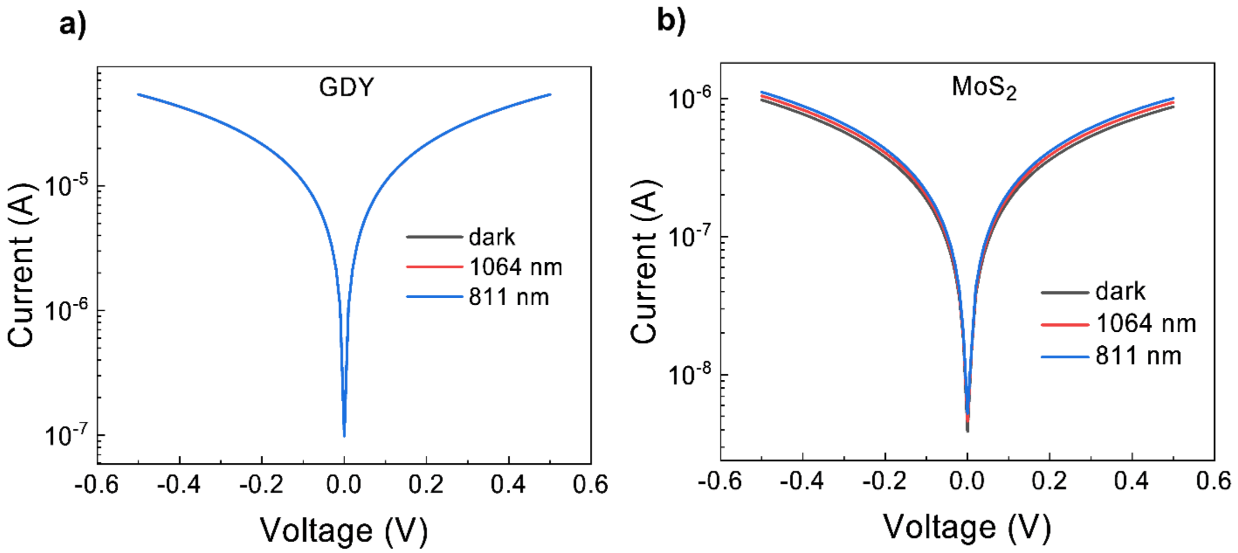

Figure S11. Figure S11. I-V characteristic of a) GDY and b) MoS_2 under 811nm and 1064 nm laser illumination.

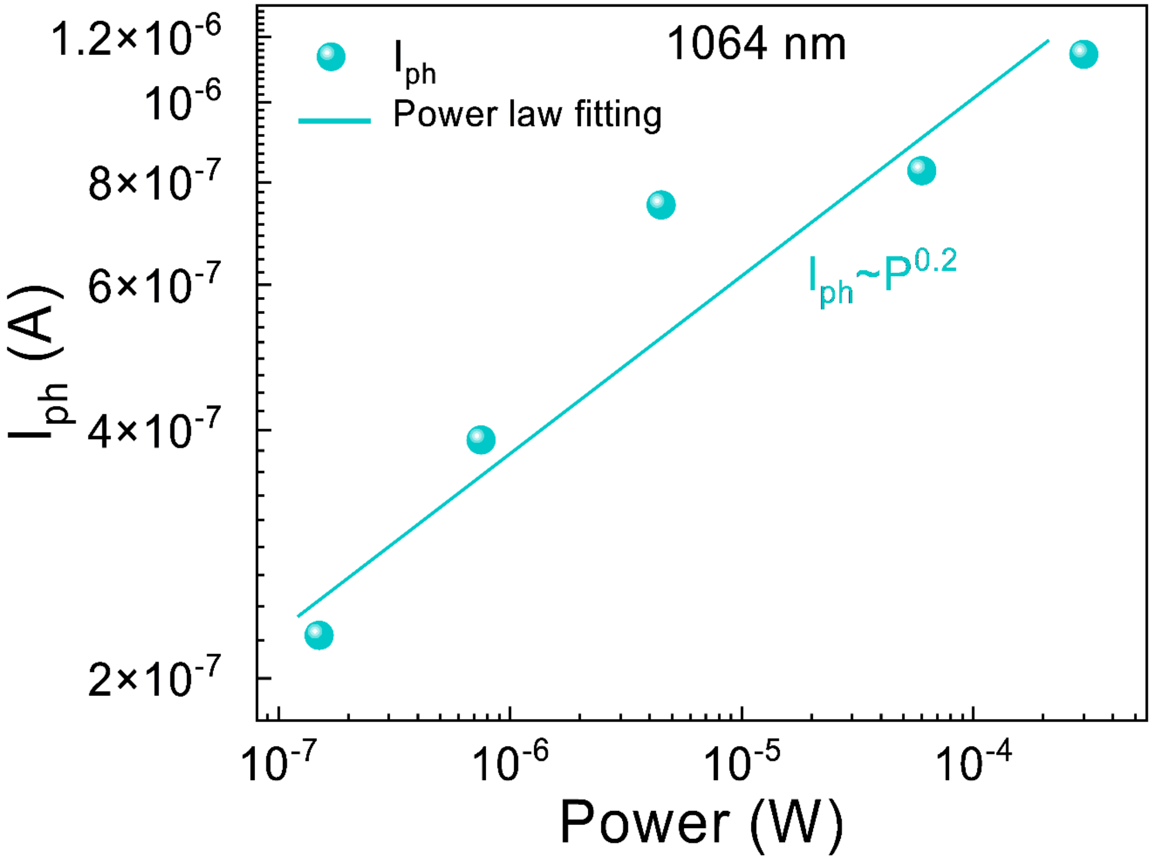

Figure S12. Power-dependent photocurrent I_{ph} under 1064 nm laser source**.**

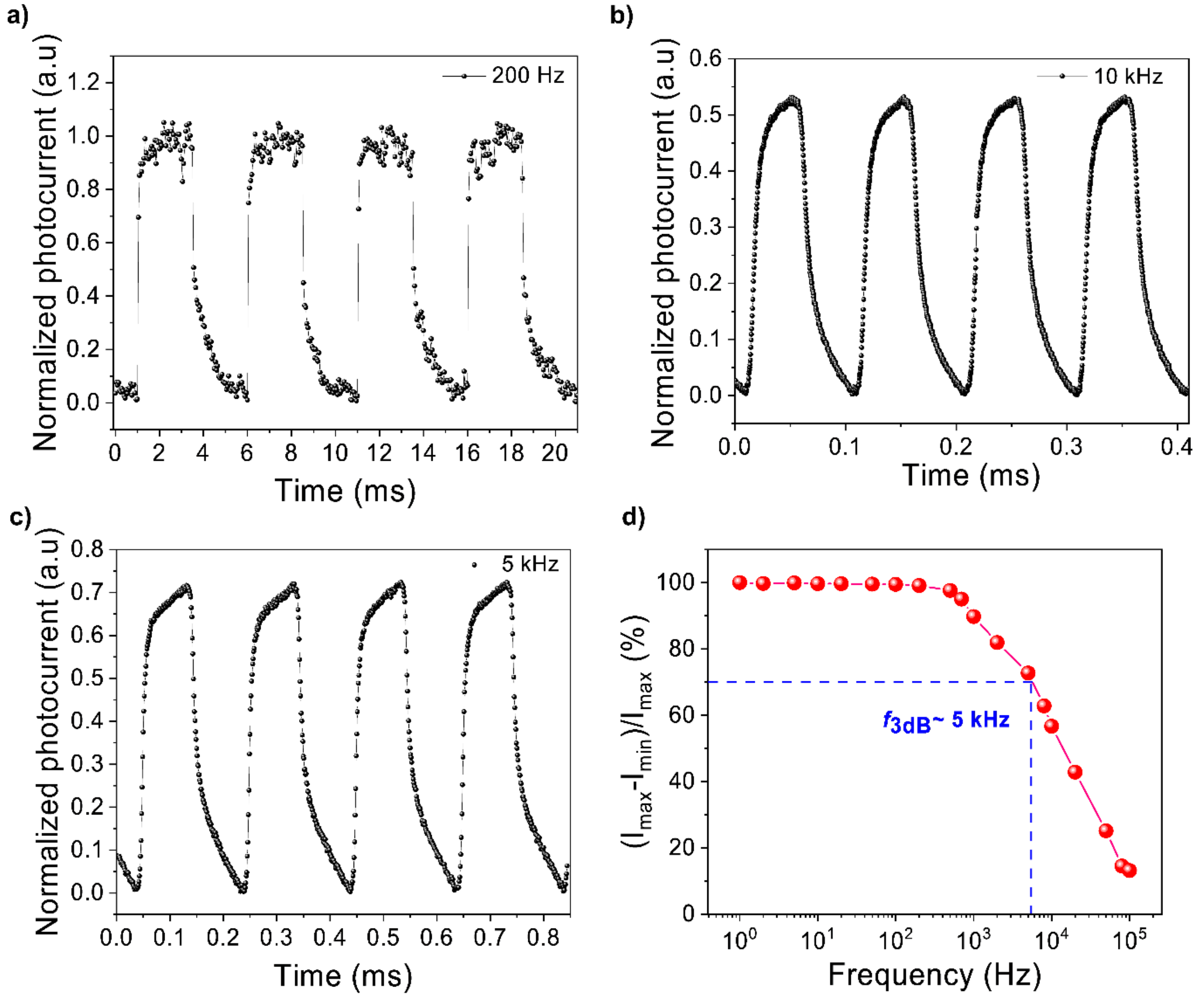

Figure S13. Frequency-dependent photoresponse ranging from 1 to 100 kHz at fixed V_d = - 0.5 V. a-c) Normalized photoresponse at 200 Hz, 10 kHz, and 5 kHz, respectively. d) The relative balance of $[(I_{max}-I_{min})/I_{max}]$ as function of frequency.

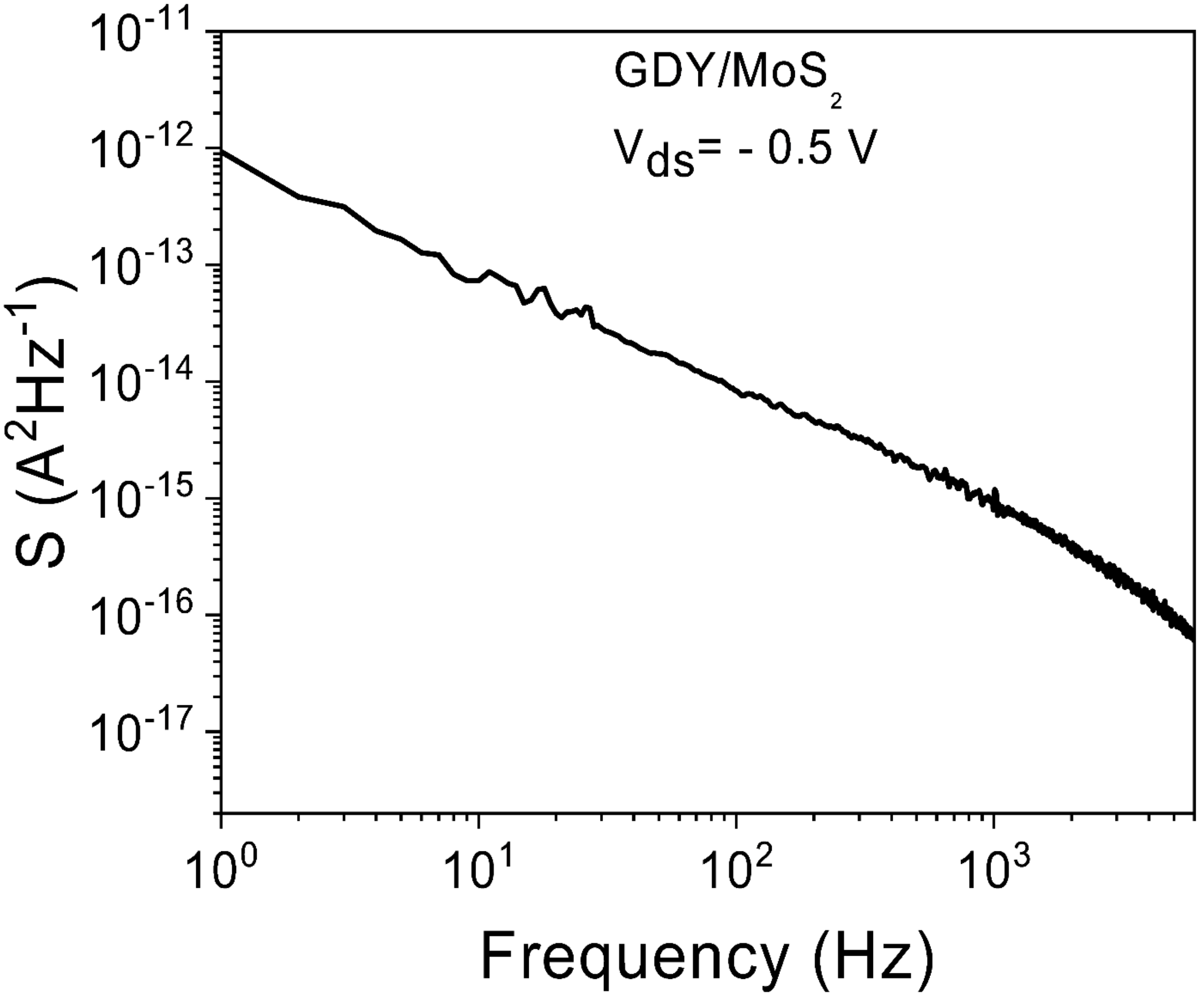

Figure S14. Noise spectral density of GDY/MoS_2 device at V_{ds}= - 0.5 V.

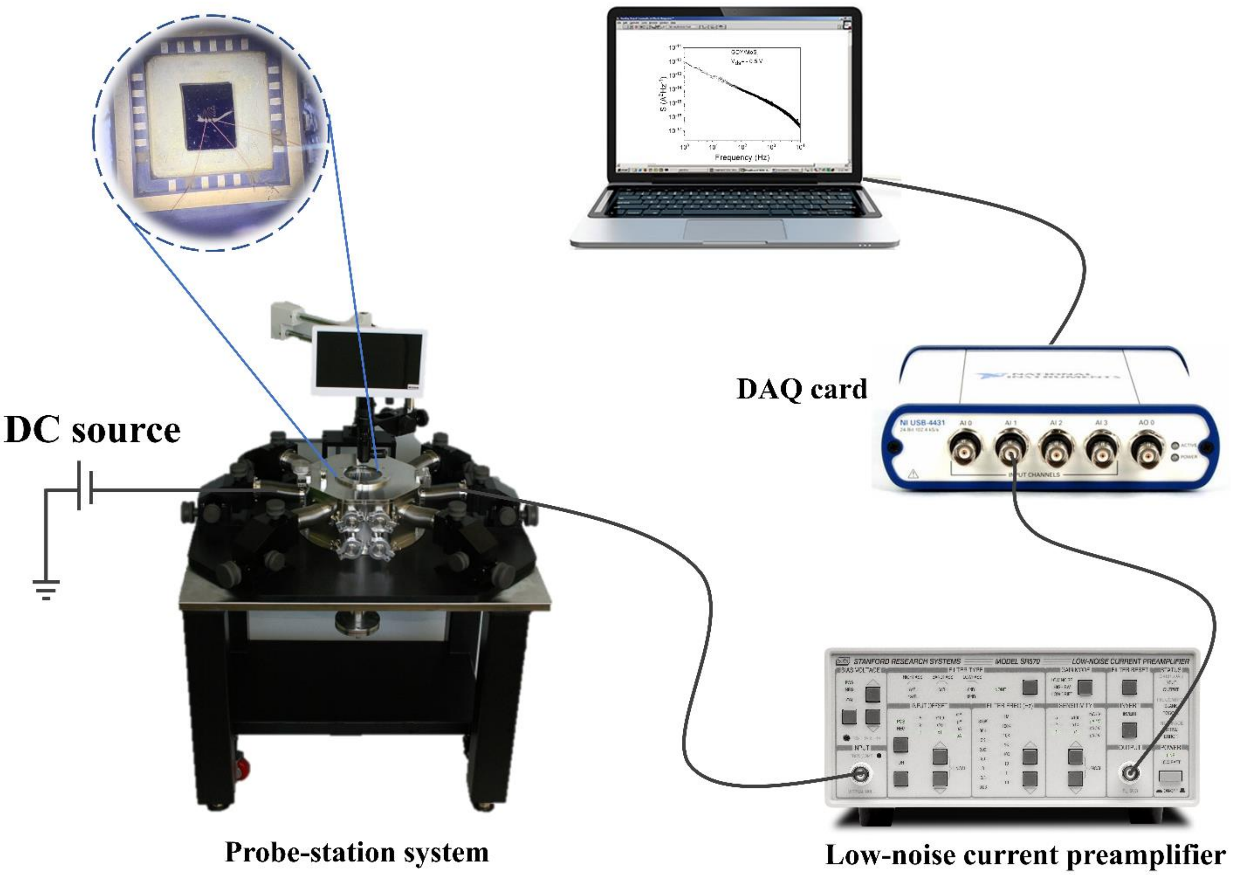

Figure S15. The schematic set-up for Low-noise current.

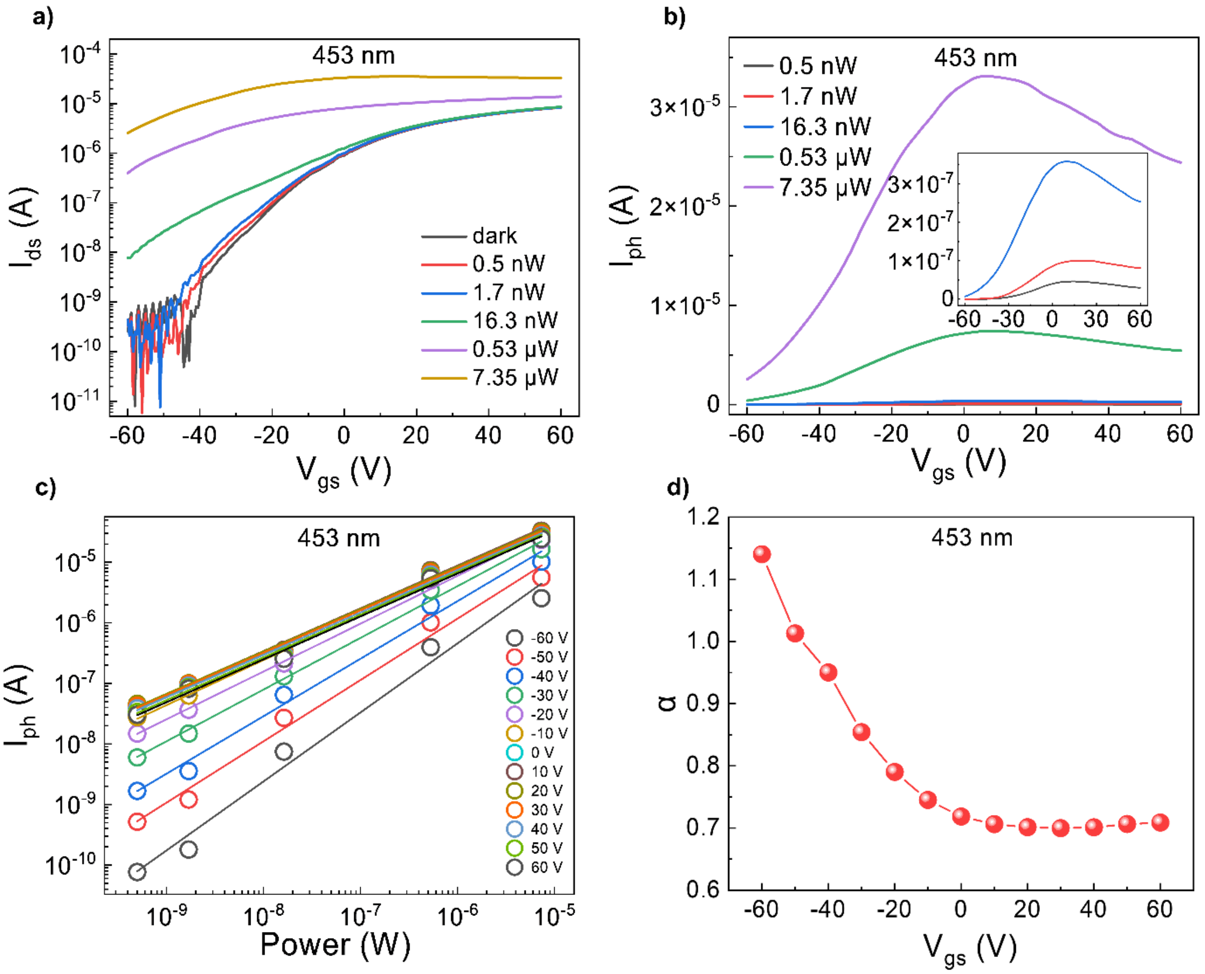

Figure S16. Gate-dependent photobehaviour of GDY/MoS_2 under 453 nm laser irradiation at fixed V_{ds} = - 0.5 V. a) Transfer curves at various incident power. b) Extracted power as function of V_{gs}. Inset: Enlarge I_{ph} at 0.5 nW and 1.7 nW. c) Extracted photocurrent as function of power. d) The extracted α value as function of V_{gs}.

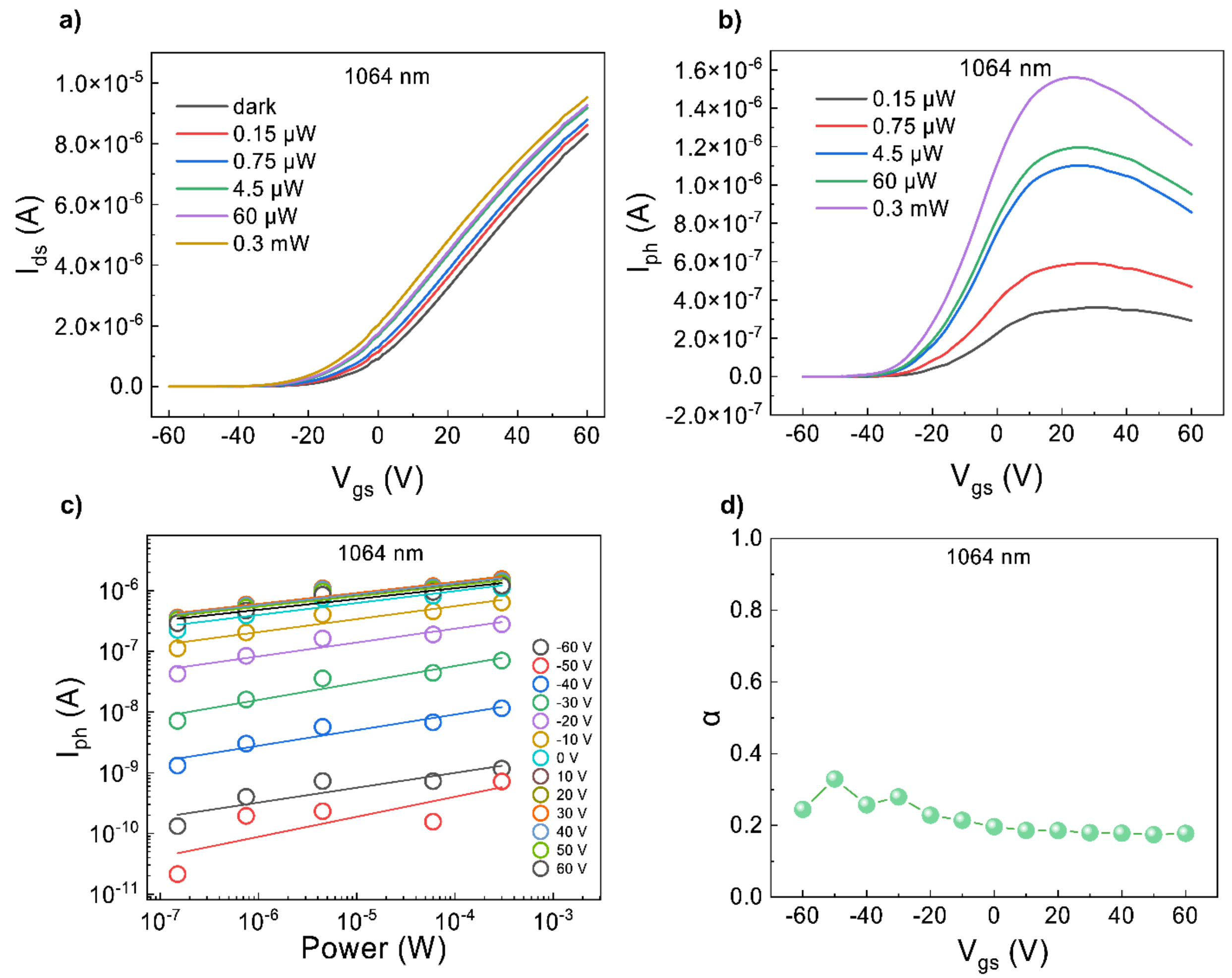

Figure S17. Gate-dependent photobehaviour of GDY/MoS_2 under 1064 nm laser irradiation at fixed V_{ds} = - 0.5 V. a) Transfer curves at various incident power. b) Extracted power as function of V_{gs}. c) Extracted photocurrent as function of power. d) The extracted α value as function of V_{gs}.

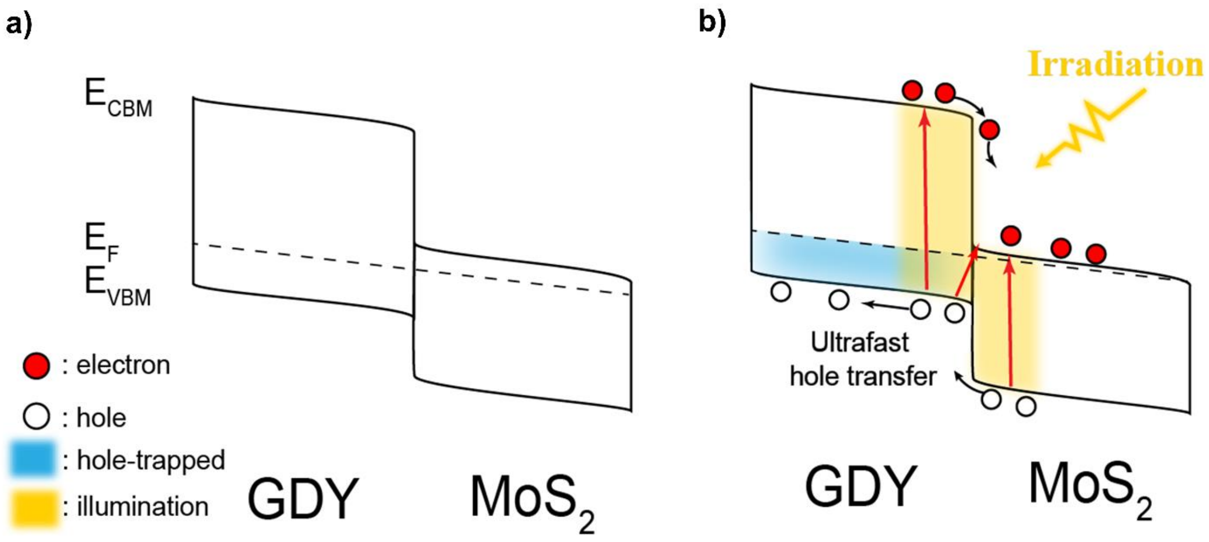

Figure S18. Energy band diagram of GDY/MoS_2 a) before and b) after illumination.

REFERENCE

[1] R. Gherabli, S. Indukuri, R. Zektzer, C. Frydendahl, U. Levy, *Light: Sci. Appl.* **2023**, *12*, 60.

[2] K. Y. Thai, I. Park, B. J. Kim, A. T. Hoang, Y. Na, C. U. Park, Y. Chae, J. H. Ahn, *ACS Nano* **2021**, *15*, 12836.

[3] G. Wu, X. Wang, Y. Chen, S. Wu, B. Wu, Y. Jiang, H. Shen, T. Lin, Q. Liu, X. Wang, P. Zhou, S. Zhang, W. Hu, X. Meng, J. Chu, J. Wang, *Adv Mater* **2020**, *32*, 1907937.

[4] J. Chen, L. Li, P. Gong, H. Zhang, S. Yin, M. Li, L. Wu, W. Gao, M. Long, L. Shan, F. Yan, G. Li, *ACS Nano* **2022**, *16*, 7745.

[5] K. Zhang, X. Fang, Y. Wang, Y. Wan, Q. Song, W. Zhai, Y. Li, G. Ran, Y. Ye, L. Dai, *ACS Appl. Mater. Interfaces* **2017**, *9*, 5392.

[6] P. Luo, K. Pei, F. Wang, X. Feng, H. Li, X. Liu, J. Luo, T. Zhai, *Sci. China Mater.* **2021**, *64*, 3017.

[7] L. Alei, C. Qianxue, W. Peipei, G. Yuan, Q. Tailei, W. Peng, T. Fangdong, Z. W. Judy, C. Rui, Z. Liyuan, G. Youpin, *Adv. Mater.* **2019**, *31*, 1805656.

[8] G. Xu, D. Liu, S. Li, Y. Wu, Z. Zhang, S. Wang, Z. Huang, Y. Zhang, *Nano Res.* **2021**, *15*, 2689.

[9] J. Yuan, T. Sun, Z. Hu, W. Yu, W. Ma, K. Zhang, B. Sun, S. P. Lau, Q. Bao, S. Lin, S. Li, *ACS Appl. Mater. Interfaces* **2018**, *10*, 40614.

[10] H. J. Jin, C. Park, K. J. Lee, G. H. Shin, S. Y. Choi, *Adv. Mater. Technol.* **2021**, *6*, 2100494.

[11] Z. Long-Hui, L. Sheng-Huang, L. Zhong-Jun, Z. Zhi-Xiang, Z. Teng-Fei, X. Chao, M. Chun-Hin, C. Yang, L. Shu Ping, L. Lin-Bao, T. Yuen Hong, *Adv. Funct. Mater.* **2018**, *28*, 1705970.

[12] K. E. Chang, C. Kim, T. J. Yoo, M. G. Kwon, S. Heo, S. Y. Kim, Y. Hyun, J. I. Yoo, H. C. Ko, B. H. Lee, *Adv. Electron. Mater.* **2019**, *5*, 1800957.

[13] G. Zhiwei, J. Weifeng, Z. Yu, D. Yu, Y. Bin, L. Chu, X. Wanjin, L. Yanping, P. Hailin, L. Zhongfan, D. Lun, *Nanoscale* **2013**, *5*, 5576.

[14] T. Shen, F. Li, Z. Zhang, L. Xu, J. Qi, *ACS Appl. Mater. Interfaces* **2020**, *12*, 54927.

[15] X. Yu, P. Yu, D. Wu, B. Singh, Q. Zeng, H. Lin, W. Zhou, J. Lin, K. Suenaga, Z. Liu, Q. J. Wang, *Nat. Commun.* **2018**, *9*, 1545.
